# Adaptation in Toxic Environments: Arsenic Genomic Islands in the Bacterial Genus *Thiomonas*

**DOI:** 10.1371/journal.pone.0139011

**Published:** 2015-09-30

**Authors:** Kelle C. Freel, Martin C. Krueger, Julien Farasin, Céline Brochier-Armanet, Valérie Barbe, Jeremy Andrès, Pierre-Etienne Cholley, Marie-Agnès Dillies, Bernd Jagla, Sandrine Koechler, Yann Leva, Ghislaine Magdelenat, Frédéric Plewniak, Caroline Proux, Jean-Yves Coppée, Philippe N. Bertin, Hermann J. Heipieper, Florence Arsène-Ploetze

**Affiliations:** 1 Laboratoire Génétique Moléculaire, Génomique et Microbiologie, UMR7156, CNRS-Université de Strasbourg, Département Microorganismes, Génomes, Environnement, Equipe Ecophysiologie Moléculaire des Microorganismes, Institut de Botanique, Strasbourg, France; 2 Department Environmental Biotechnology, Helmholtz Centre for Environmental Research–UFZ, Leipzig, Germany; 3 Université de Lyon, Université Lyon 1, CNRS, UMR5558, Laboratoire de Biométrie et Biologie Évolutive, Villeurbanne, France; 4 Laboratoire de Biologie Moléculaire pour l’Etude des Génomes, (LBioMEG), CEA-IG-Genoscope, Evry, France; 5 Plate-Forme Transcriptome et Epigénome, Centre d'Innovation et de Recherche Technologique—Département Génomes et Génétique, Institut Pasteur, Paris, France; 6 Université de Haute-Alsace, Biopôle–LVBE, Colmar, France; National Environmental Engineering Research Institute CSIR, INDIA

## Abstract

Acid mine drainage (AMD) is a highly toxic environment for most living organisms due to the presence of many lethal elements including arsenic (As). *Thiomonas (Tm*.*)* bacteria are found ubiquitously in AMD and can withstand these extreme conditions, in part because they are able to oxidize arsenite. In order to further improve our knowledge concerning the adaptive capacities of these bacteria, we sequenced and assembled the genome of six isolates derived from the Carnoulès AMD, and compared them to the genomes of *Tm*. *arsenitoxydans* 3As (isolated from the same site) and *Tm*. *intermedia* K12 (isolated from a sewage pipe). A detailed analysis of the *Tm*. sp. CB2 genome revealed various rearrangements had occurred in comparison to what was observed in 3As and K12 and over 20 genomic islands (GEIs) were found in each of these three genomes. We performed a detailed comparison of the two arsenic-related islands found in CB2, carrying the genes required for arsenite oxidation and As resistance, with those found in K12, 3As, and five other *Thiomonas* strains also isolated from Carnoulès (CB1, CB3, CB6, ACO3 and ACO7). Our results suggest that these arsenic-related islands have evolved differentially in these closely related *Thiomonas* strains, leading to divergent capacities to survive in As rich environments.

## Introduction

Recent studies revealed that both horizontal gene transfer (HGT) and Genomic Islands (GEIs) may confer selective advantages to bacteria, and are essential in adaptation to extreme environments [1–3]. GEIs consist of discrete DNA segments (ranging from 10 to 200 kbp in size) which sometimes differ in their nucleotide features (G+C content or codon usage) from the rest of the genome, and have often been found in the vicinity of tRNA or tRNA-like genes. The boundaries of these islands frequently correspond to perfect or near-perfect direct repeats (DRs). These regions often harbor functional or cryptic genes encoding integrases originated from phages or genes involved in plasmid conjugation processes. GEIs thus include elements of other kinds such as integrative and conjugative elements (ICE), conjugative transposons and cryptic or defective prophages and can result from one or several HGT events and genomic rearrangements [2]. Thus, studying bacterial colonization of diverse niches is one way to understand the forces driving bacterial evolution [4–6], which plays a particularly important role in habitats with high levels of toxic metals that provide stressful conditions. These extreme habitats can arise naturally, or as a result of human activities, although in both cases, bacteria have adapted to thrive in these challenging ecological niches. Acid mine drainage (AMD) is an excellent example of a stressful environment in which only few organisms can survive. Mining activities, involving the processing of sulfide ores, lead to the production of toxic, metal rich waste, and when mines are left exposed, weathering of the ores results in AMD [7]. Ultimately, AMD contaminates runoff and produces streams with acidic pH and high concentrations of heavy metals. Common pollutants include arsenite (As(III)) and arsenate (As(V)), two inorganic forms of arsenic (As) which are highly toxic and dramatically impact microbial community composition. The Carnoulès site in the south of France has a typical AMD profile with a pH of approximately 3 and particularly high levels of As, ranging in concentration from 50 to 350 mg/L. This AMD is therefore an excellent model site to examine extreme microbial environments and has been the focal point of a suite of studies [8–11].

Bacteria belonging to the genus *Thiomonas* are found ubiquitously at AMD sites and several strains (including CB1, CB2, CB3, CB6 and 3As), have been isolated from the Carnoulès AMD [12–16]. These facultative chemolithoautotrophic betaproteobacteria are able to use reduced inorganic sulfur compounds (RISCs) as electron donors. *Tm*. *delicata* (previously *Tm*. *arsenivorans* b6) is capable of autotrophic growth while *Tm*. spp. CB1, CB2, CB3 and CB6 survive only in the presence of organic carbon sources [13–15]. Using Comparative Genomic Hybridization (CGH), our previous work demonstrated that *Thiomonas* genomes have been shaped by the acquisition and loss of GEIs, which may have helped strains adapt to different niches within the AMD [16]. While it was possible to detect GEIs and uncover evidence of HGT using the CGH approach, forces that lead to bacterial adaptation in toxic environments are not thoroughly understood. The genome of two *Thiomonas* strains, *Thiomonas arsenitoxydans* 3As (3As) isolated from the Carnoulès AMD [16], and *Thiomonas*. *intermedia* K12 (K12) isolated from sewage pipes [17], (http://genome.jgi-psf.org/thiin/thiin.info.html) are available in public databases and provided the initial glimpse into the genetic capacity of these *Thiomonas* strains. In particular, *Tm*. *intermedia* K12 has not been well studied and, to our knowledge, its phylogenetic relationship with other characterized *Thiomonas* strains, in particular with the *Tm*. *arsenitoxydans* strain 3As was never analyzed. The DNA-DNA hybridization values proposed suggest that while it clearly belongs to a different species, 3As is genetically related to the *Tm*. *intermedia* strain, DSM 18155 [18]. The isolates designated as 3As, CB1, CB2, CB3 and CB6 are phylogenetically related and share more than 99% sequence ID according to the 16S rRNA [16]. Despite their relatedness, these isolates each have unique metabolic capacities [15,16,19], thus representing an ideal model system to analyze bacterial genomic divergence in extreme and polluted environments on a fine evolutionary scale.

In this study, we sequenced the genome of strain CB2, and optimized the assembly in order to compare this genome’s architecture to that of 3As and K12. Our results revealed that the CB2 genome was subject to rearrangement and contains genomic islands involved in heavy metal transformation and resistance. Second, we sequenced the genomes of additional five *Thiomonas* strains isolated from AMD and we compared those draft genomes with the genomes of CB2, 3As and K12. This revealed that the *Thiomonas* strains that originated from the AMD have adapted to an extremely hazardous habitat by maintaining specific GEIs that confer As resistance as well as the capacity to oxidize arsenite.

## Materials and Methods

### Bacterial cultivation and growth conditions

In this study, the strains *Thiomonas* spp. ACO3, ACO7 were isolated on R2A agar medium (BD Difco) after 7 days of incubation at 30ºC from sediments collected at the Carnoulès AMD after being processed on a Nycodenz gradient as previously described [20]. The strains *Thiomonas* spp. CB1, CB2, CB3, CB6, and 3As (DSM 22701) were previously isolated from AMD-impacted water at the Carnoulès AMD (France) [14,16] and K12 was obtained from a corroded concrete wall of the Hamburg sewer system [17]. After isolation or recovery from cryogenic stocks (for all strains previously isolated) the *Thiomonas* spp. studied here were maintained at 30°C in modified m126 medium. The composition in one liter of diH_2_O was: 4.5 g Na_2_HPO_4_; 1.5 g KH_2_PO_4_; 5.0 g Na_2_S_2_O_3_; 0.3 g NH_4_Cl; 0.1 g MgSO_4_.7H_2_0; and 0.5 g yeast extract. The pH of the media was adjusted to 5.0 with H_2_SO_4_. Variations of this media included the addition of sodium arsenite (from a 500 mM stock solution), at the concentration listed in the text. *Klebsiella pneumoniae ozenae* KIIIA [21] was used in the plasmid visualization experiment, and was grown in LB medium (BD Difco) at 30°C.

### Assessment of arsenite oxidation

In an initial set of experiments, 100 mL of either standard m126 or m126 supplemented with 5 g/L glucose was inoculated with CB2 from a liquid preculture to an OD_600_ ~ 0.002 and then was incubated at 30°C while horizontally shaking at 120 rpm. After 24 hours of unimpeded growth, As(III) was added from a sterile stock to a final concentration of 3 mM. Uninoculated flasks with the respective media served as abiotic controls. Samples of 1 mL were taken at daily intervals and centrifuged for 10 minutes at 16, 000 x *g*. After centrifugation, the supernatants were stored at 4°C in order to avoid precipitation of dissolved As salts observed in frozen samples. For speciation analysis, all samples were diluted 100-fold with distilled water before being subjected to inductively coupled plasma mass spectrometry (ICP-MS) or inductively coupled plasma atomic emission spectroscopy (ICP-AES) analysis. Standards of inorganic As(III) and As(V) were used for calibration throughout all experiments. In a second set of experiments, three independent 25 mL cultures of m126 supplemented with 1.33 mM of As(III) were inoculated with CB1, CB2, CB3, CB6, K12 or 3As to an OD_600_ ~ 0.002. An abiotic control (no inoculation) was also prepared. These cultures were incubated for 120 h at 30°C shaking at 120 rpm. 600 μL of the cultures were sampled at 0 h, 24 h, 48 h, 72 h, and 120 h of growth and were immediately centrifuged at 10, 000 x *g* for 10 min, after which 500 μL of the supernatant was diluted in 12 mL of ultrapure water. This dilution was then injected in an anion-exchange chromatography column (BOND ELUT JR-SAX, 500MG, Agilent Technologies) which retained the As(V), while the flow through fraction contained As(III). The bound As(V) was eluted with 0.12 M HCl solution. The two As(III) and As(V) fractions obtained were conserved at 4°C, and the As concentration was assessed by ICP-AES.

### Determination of arsenite minimal inhibitory concentration (MIC)

20 mL of m126 was inoculated with K12, CB1, CB2, CB3, CB6, 3As, ACO3, or ACO7 to an OD_600_ ~ 0.002 and incubated for 40 h at 30°C, with shaking at 120 rpm. After growth, the cultures were diluted to an OD_600_ ~ 0.1 and serial dilutions were then made in m126. Then, 2 μL of each dilution was spotted on solid m126 plates supplemented with different concentrations of arsenite (1.33 mM and from 6.67 to 16 mM), and incubated at 30°C for 10 days.

### Western blot

Monoclonal antibodies raised against the AioA peptide were obtained from Perbio Science (Erembodegem, Belgium). Briefly, a peptide with the sequence CGYHAYTWDADREGGRAPHC was synthesized. This peptide corresponds to the N-terminal amino acids residues 29 to 47 of the arsenite oxidase large subunit of *Tm*. *arsenitoxydans* 3As. The peptide was then coupled to keyhole limpet haemocyanin (KLH). Two rabbits were injected with peptide-KLH and bled at day 90. Antibodies were partially purified on an affinity column substituted with the peptide. Excess antibodies were removed by repeated washing with PBS and then 50 mL of a stationary phase culture in m126 was prepared containing 0, 0.67, 1.33 or 2.66 mM As(III) and centrifuged at 7, 000 x *g*. The pellets were suspended in Laemmli buffer [22] and boiled for 1 min. 15 μL of these extracts were separated by SDS-PAGE using a Amersham^TM^ ECL^TM^ gel 4–12% (GE Healthcare). After SDS-PAGE electrophoresis, the proteins were electrotransfered to a PVDF low-fluorescent membrane (Thermo Scientific) using a TE77 Semi-dry transfer unit (Amersham Biosciences) at 45 mA for 1 h according to the manufacturer’s instructions. The membranes were blocked in SuperBlock^®^ blocking buffer (Thermo Scientific) and then washed three times in PBS2 buffer (80 mM Na2HPO4, 20 mM NaH2PO4, 100 mM NaCl, 0.01% tween 20) and incubated for 1 h with AioA antisera (1:1, 000 dilution) in PBS. After a 1 hour incubation in PBS containing the secondary antibody (1:5, 000 dilution of goat anti-rabbit IgG DylightTM 488 conjugated highly cross-adsorbed, Thermo Scientific), the membrane was washed twice with PBS in the dark and assessed using a Typhoon Scanner (GE Healthcare).

### Sequencing, annotation and optical map analysis of the *Thiomonas* spp. genomes

The 3As genome was previously sequenced and described [16], while the K12 genome was sequenced by the US DOE Joint Genome Institute (NC_014153.1, NC_014154.1, NC_014155.1). The genomes analyzed for this study were integrated into the MicroScope platform [23–25]. CB1, CB2, CB3, CB6, ACO3, and ACO7 genomic DNAs were prepared using the Wizard genomic kit (Promega). The *Thiomonas* CB2 genome was sequenced *de novo* by MWG operon (http://www.operon.com/) using Roche 454 Sequencing through the use of shotgun and paired end (8 kb in size) libraries. The 425, 947 reads gave a 28x coverage. The assembly with Newbler (454 Life Science/Roche) yielded 92 contigs and ultimately 16 scaffolds. To optimize the CB2 assembly and to compare it to that of K12 and 3As, an optical map was generated using the Argus™ Whole-Genome Mapping System (www.opgen.com, Gaithersburg, Maryland, USA). The optimal restriction enzyme *Bgl*II was chosen using “Enzyme Chooser” (OpGen Inc., Gaithersburg, MD), a software program allowing for the identification of the best enzyme for analysis. The selected enzyme produced an average fragment 6–12 kb in size with no single restriction fragment larger than 80 kb across the genome. Overall, 180 single-molecule restriction maps with an average size of 214 kb each were generated. The assembly of different molecules permitted us to obtain a unique circular map (3.5 Mb). Contig fasta files were imported into MapSolver™ software and *in silico* maps were created using *Bgl*II restriction sites. The *in vitro* and *in silico* maps were compared for each of the 16 scaffolds, enabling their reorganization into 9 scaffolds. The largest scaffold spanned 3.8 Mb, which was over 98% of the CB2 genome sequence. Accession numbers for the CB2 genome (EMBL database) are from LK931581 to LK931672.

The CB1, CB3, CB6, ACO3 and ACO7 genomes were sequenced on an Illumina instrument (www.illumina.com). Genomic DNA of the strains CB1, CB3, and CB6 was fragmented and inserts approximately 8 kb in length were selected to construct mate pair indexed libraries. These libraries were loaded on a MiSeq sequencing device flowcell and sequenced on pair-ends, 150 nt length. CB1, CB3, and CB6 reads were assembled in parallel using Velvet (De Bruijn Graph) (https://www.ebi.ac.uk) and using Newbler (OLC) (www.roche.com, Basel, Switzerland) followed by SSPACE [26] to organize the contigs longer than 500 nt. The different assemblies were compared and the best were retained (performed with Newbler for CB1 and CB6 and with Velvet for CB3). The gap filling was completed for the 3 strains using GapCloser (http://soap.genomics.org.cn/soapdenovo.html) on scaffolds more than 2 kb. Genomic DNA of ACO7 and ACO3 were fragmented and inserts between 500 and 600 nt were selected to construct pair-end indexed libraries. These libraries were loaded on a MiSeq sequencing device flowcell and 300 nt length sequences were obtained for each extremity. The data were merged before assembly with Newbler and in order to reduce the number of undetermined bases, GapCloser was used. Due to the high similarities of sequence between these genomes and the reference strain 3As genome, contigs longer than 500 nt were then organized by comparison with 3As genome sequence. Accession numbers for the CB1, CB3, CB6, ACO3 and ACO7 genomes are LN831666-LN831688, LN831730-LN831775, LN831689-LN831714, CTRK01000001-CTRK01000065 and CTRL01000001-CTRL01000068, respectively (EMBL database).

The automated annotation was completed by Genoscope (Centre national de séquençage français) with the MicroScope platform [23–25]. MicroScope uses the Prokaryotic Genome DataBase (PkGDB), to analyze and compare the annotation of genomes of bacteria and Achaea. A manual annotation was completed for genes in the CB2 genomic islands RGP10, RGP19, and RGP9. The data and annotations are accessible using the MicroScope web interface, MaGe (Magnifying Genomes). Whole genome comparisons were performed using the nucmer module of the MUMmer 3.0 program [27] with default parameters. Dot plot figures were subsequently generated using mummerplot with the color parameter on. RGP finder, a module in MaGe [25] was used to initially identify and compare the genomic islands of *Thiomonas* strains. Comparative genomic analyses were performed using the MicroScope plateform tools (“Pan/core-genome” option) using the default MICFAM parameters.

### Visualization of plasmids

Plasmids were visualized using the Wheatcroft’s method method [28] with some modifications. *Klebsiella pneumoniae ozenae* KIIIA [21] was grown at 30°C in LB. This strain contains 3 plasmids with estimated sizes of 225 kb, 130 kb and 45 kb [29]. *Thiomonas* strains were grown at 30°C in m126 medium. For each strain, a volume of culture equivalent to 1.5 mL of cells at OD_620nm_ = 0.4 was harvested and centrifuged for 3 min at 13, 000 x *g* and 4°C. The pellet was suspended in 0.5 mL water at 4°C. The cell suspension was layered onto 1 mL of 0.3% N-lauroylsarcosine sodium salt solution and centrifuged at 13, 000 x *g* for 3 min at 4°C. The pellet was immediately resuspended in 40 μL of 40 mM Tris, 10 mM EDTA, 20% Ficoll and incubated on ice for 15 min. A 0.75% agarose gel (pulse field certified agar, Bio-Rad) was prepared with TBE buffer and 25 μL of 10% SDS containing xylene cyanole was loaded in each well. Migration was completed in TBE buffer at 100 V reversing polarities of the electrodes until the dye migrated 1 cm behind the wells. 20 μL of lysis solution was added to each cell suspension and 60 μL was loaded on the gel. The lysis solution used for *K*. *pneumoniae* and *T*. *intermedia* K12 contained 100 mM Tris, 10 mM EDTA, 0.4 mg/mL RNase and 10 mg/mL lysozyme. For CB1, CB2, CB3, CB6 and 3As, 10 mg/mL proteinase K and 4 μL/mL ß-glucanase 2 (20 mg/mL) were added to the lysis solution and the lysozyme concentration was increased to 15 mg/mL. Migration was performed at 50 V for 30 min and then at 100 V for 5 h. The gel was stained with ethidium bromide and visualized under a UV-transilluminator.

### PCR experiments

Cells were grown in m126 medium for 48h and centrifuged at 5, 000 x *g* for 15 minutes. DNA was extracted as previously described [15]. The presence of the two plasmids was verified by PCR using a Taq Core Kit (MP Biomedicals). Each reaction mixture (total volume of 25μL) contained 250 ng of DNA. Thermocycling conditions were as follows: 2 minutes at 94°C followed by 35 cycles of 40 seconds at 94°C, 45 seconds at 61°C and 4 minutes at 72°C. A final elongation step at 72°C for 7 min was added. Primers were designed as follows: 870001_280sor 5’-CAGCGATGTGCACATCGAGC-3’, 870008_10220sor 5’-GCAGTAGCTCGCGGATGATG-3’, 880002_180sor 5’-CGTTCTAGGCGTACCGGTAC-3’, 8800035_20340sor 5’-CAGCGCAAGTCTTCCTGGTC-3’, 890001_145sor 5’-CGATCGTACTGGGTGAGCTC-3’, 890016_7140sor and 5’-GAGCCTGAGCGTGAGCAATC-3’ for the ascertainment of the two plasmids and 730161-830for 5’-GCCTGCTGTACGAAGCCAAC-3’, 730162-1610rev 5’-CCACGATCAGGTCGACAACC-3’, 780013-120for 5’-GCGGCTAAGTCTGAGGAACG-3’ and 780014-850rev 5’-CCGTCGGATGTGTCGATGTG-3’ for testing the circularization of the ICE19. Products of amplification were sequenced at Eurofins Genomics.

### RNA sequencing

CB2 was cultivated in 20 mL of m126 liquid medium until OD_600_ = 0.2. Cells were then diluted 12.5-fold in the same medium in the absence or presence of 1.3 mM As(III) and incubated in a 6-well plate (Costar, Corning, USA) without shaking. After 48 h the planktonic cells were carefully removed and new sterile medium was added. After 72 h the planktonic cells were removed, biofilm cells were washed 2-times with NaCl 9 g/L and were manually recovered by scratching the dishes with NaCl 9 g/L. Cells were pelleted by centrifugation for 15 min at 4, 000 x *g*. RNA was extracted from these pellets as previously described [30]. For RNA-Seq, enriched mRNA was obtained from 10 μg of total RNA using the rRNA capture hybridization approach from the RiboZero kit (Epicentre, Singapore), according to the manufacturer's instructions. For high-throughput sequencing, non-directional cDNA libraries were prepared from enriched fragmented mRNA using the RNA sample preparation kit, set A (Illumina, San Diego, CA, USA). Fragments of cDNA of ± 150 bp, ligated with Illumina adapters and amplified per PCR, were purified from each library. Quality and quantity was confirmed on a Bioanalyzer (Agilent, Santa Clara, CA, USA). Sequencing of 51 bases was performed in single-end mode, using an Illumina HiSeq2000 instrument (Illumina). Reads were cleaned from the adapter sequences and from sequences of low quality using an in-house program. Only sequences with a minimum length of 25 nt were considered for further analysis.

The Bowtie program was used to align the reads to the *Thiomonas* CB2 genome. Statistical analyses were performed with R software [31] and Bioconductor packages [32]. Normalization and differential analysis were carried out according to the DESeq model and package [33]. A Benjamini and Hochberg (BH) [34] p-value adjustment was performed to take into account multiple testing and control the false positive rate to a chosen level, α set to 0.05, yielding 42 up-regulated genes, i.e. more expressed in the presence of As than in the absence. In order to compare expression levels between different genes, normalized expression values were computed as follows: counts were first normalized with DESeq in order to make reads comparable between samples; these normalized counts were then divided by gene length (in nt) and multiplied by 1, 000, in order to obtain expression levels by kilobase.

## Results

### Genome sequencing of *Thiomonas* strains and assembling

**Fig 1:**
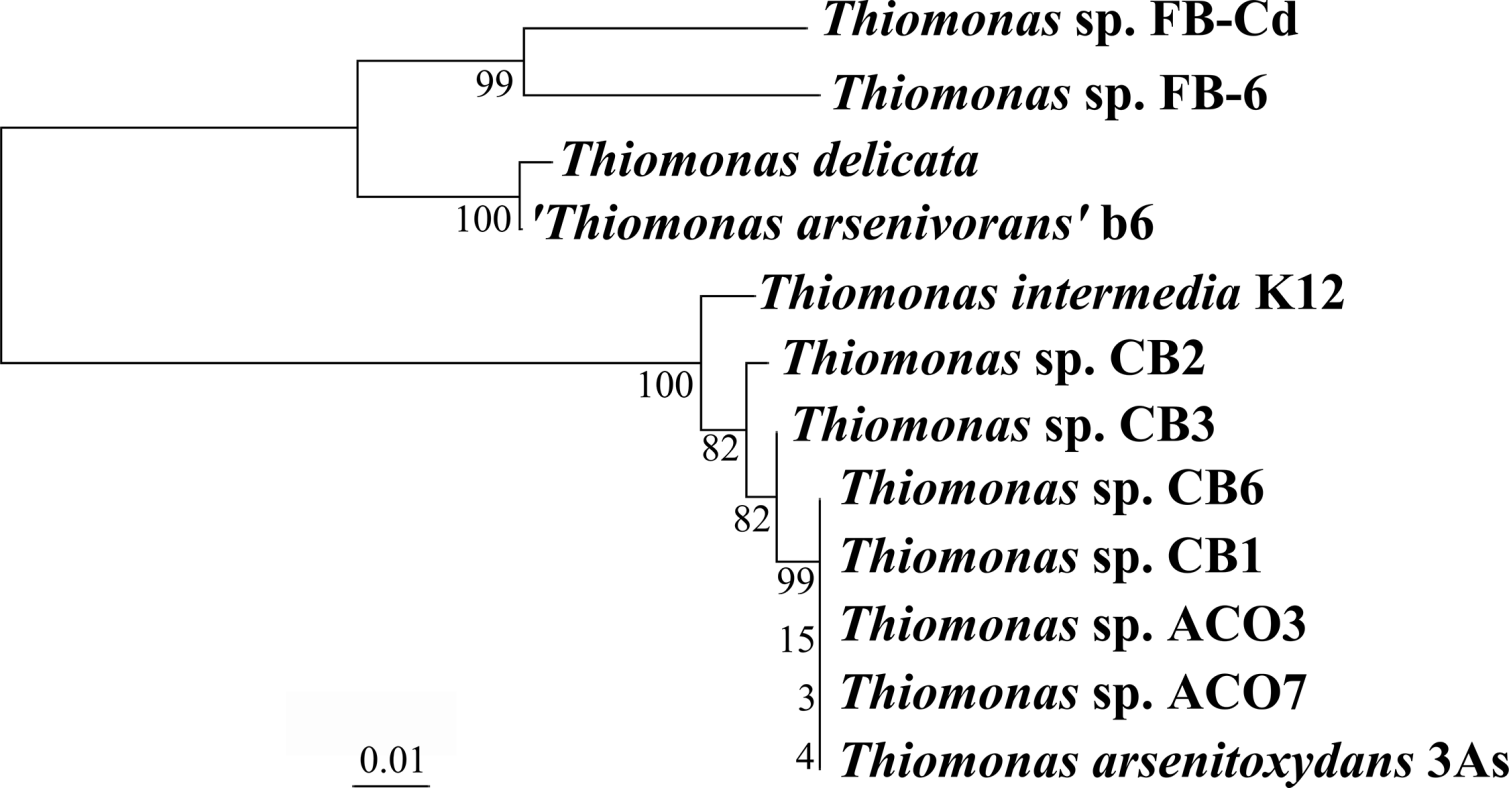
Maximum likelihood tree of the *Thiomonas* strains included in this study. The tree is based on the concatenated alignment of *rpoA*, *dnaK*, *atpD*, 16S rDNA, and 23S rDNA genes (8628 nucleotide positions). The sequences were aligned with the MUSCLE and trimmed with GBLOCKS programs implemented in SEAVIEW software version 4.5.4 [46] and then concatenated. The tree was inferred with PhyML version 3.1 [47], using the GTR+Γ4 model as suggested by the proposed model tool implemented in TREEFINDER v2011 [48], using the NNI+SPR option for topology search. Values at branch nodes correspond to bootstrap values (100 replicates) calculated with PhyML and to posterior probabilities calculated with MrBayes version 3.2.2 [49], with the GTR + G4 model. The scale bar represents the average number of substitutions per site.

There were several strains used in this study that were previously isolated from the Carnoulès AMD: 3As, CB1, CB2, CB3, and CB6. Here, we also report two novel strains isolated from this AMD community: ACO3 and ACO7. Since the phylogeny of closely related strains is difficult to resolve using only the 16S rRNA gene, we combined five markers (*rpoA*, *dnaK*, *atpD*, 16S rDNA, and 23S rDNA) to clarify the relationships among these *Thiomonas* strains (Fig 1). The resulting maximum likelihood and Bayesian trees showed that 3As, CB1, CB6, ACO3, and ACO7 are very closely related (bootstrap value (BV) = 99% and posterior probability (PP) = 1.0), and cluster with CB3 and CB2, while K12 is more divergent (BV = 82% and PP = 1.0).

The genomes of CB1, CB2, CB3, CB6, ACO3, and ACO7 were sequenced. While CB2 harbors the most divergent genome compared to 3As among these strains, we attempted to obtain the most complete genome assembly of possible for this strain. The CB2 scaffold organization was determined with an optical map, and of the 16 scaffolds, of which organization of twelve was possible. Five scaffolds could not be mapped precisely to the genome: two were too small (5 and 2.4 kb) to be assembled according to the optical map, and the other three (20.9, 10.8 and 7.9 kb) likely correspond to plasmid DNA as is indicated by the presence of *mob* and *par* genes which are involved in plasmid mobilization, replication, and stabilization [35–38]. PCR experiments were performed showing that the 7.9 Kb scaffold correspond to one small plasmid and the two other scaffolds (20.9 and 10.8 Kb) correspond to one plasmid of 31.7 Kb (data not shown). Moreover, electrophoresis of CB2 gDNA detected an extra chromosomal element less than 40 kb in length (S1 Fig), which further supports the presence of the 31.7 Kb plasmid. Similarly, one plasmid was detected in CB1 (approximately 40 kb), and two were detected in CB3 (one of approximately 40 kb and one less than 40 kb) however, no plasmid was detected in CB6 (S1 Fig). These observations are in agreement with genomic data since, in the case of CB1 and CB3, the presence of regions similar to the 3As plasmid pTHI (46.8 kb) [16] suggested that plasmids similar to pTHI were present in CB1 and CB3. These observations revealed that *Thiomonas* strains harbor various plasmids of different sizes with different sets of genes.

General features including the GC-contents (63.7%– 63.9%) are similar among the eight strains, while the genome size and gene content varied from 3.4 Mb (K12) to 4.3 Mb (CB3) (S1 Table). The CB3 genome was larger, due in part to multiple duplications (77 tandem duplications including five large duplications (>10 genes)) and had a higher percentage of repetitive sequences compared to the other genomes (S1 Table). Finally, a comparative genomic analysis of CB2, 3As, and K12 revealed that the pan genome of these three strains is comprised of 11, 016 genes, while the core-genome encompassed 7, 381 genes (80% of amino acid identity, 80% alignment coverage), representing 66.8%, 65.0%, and 69.4% of the 3As, CB2, and K12 proteomes, respectively. This analysis also highlighted that 285 genes are present in CB2 and 3As (isolates from the Carnoulès AMD,) but not in K12 (https://www.genoscope.cns.fr/agc/microscope/home/index.php). These genes encode enzymes involved in glutamate, biotin, and cobalamin biosynthesis, toxic metal resistance, nitrite and nitrate reduction, sulfate assimilation, transcriptional regulation, permeases, methyl-accepting chemotaxis proteins, transporters, as well as proteins of unknown function (https://www.genoscope.cns.fr/agc/microscope/home/index.php) representing 7.5% (CB2) to 7.8% (3As) of the AMD genomes, and possibly required for survival in AMD.

### Comparison of the genome structure of *Thiomonas* strains CB2, 3As, and K12

**Fig 2:**
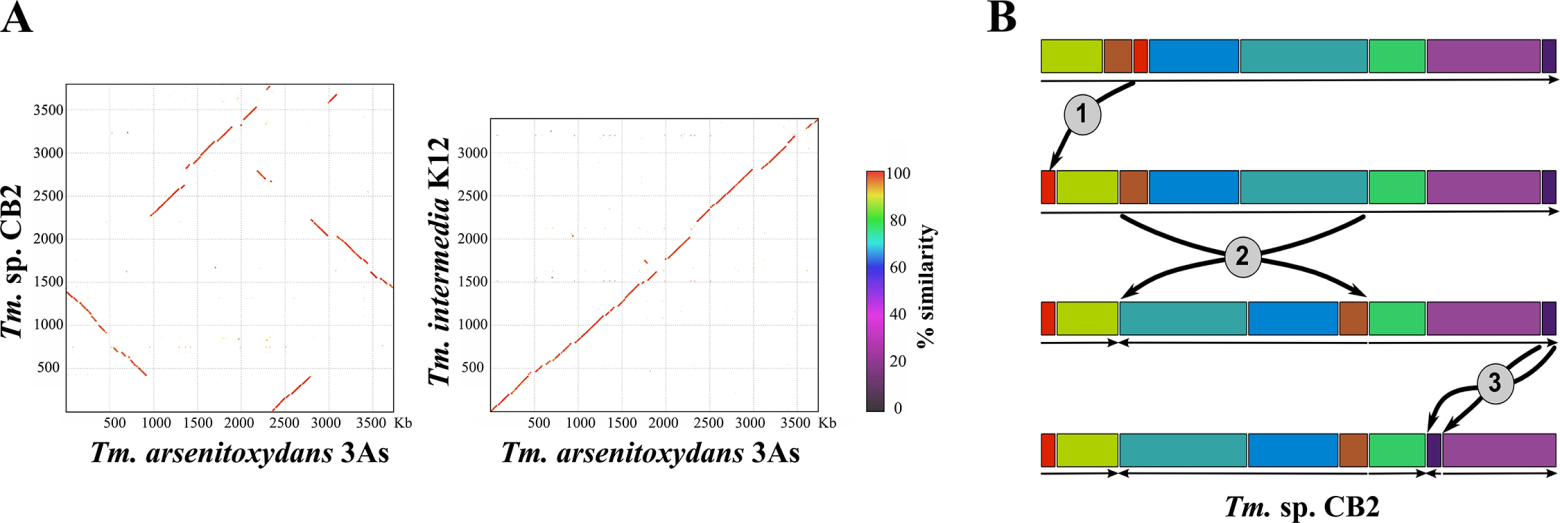
Comparison among the CB2, K12, and 3As genomes. (A) Dot plot, CB2 vs 3As and 3As vs K12. The genomes of 3As and K12 are well conserved while the CB2 genome appears to have undergone chromosomal rearrangements. (B) Scheme demonstrating the key differences between the *Thiomonas* ancestor and CB2 genome. Above, “1”, “2” and “3” highlight a translocation of 0.1 Mb, an inversion of 1.8 Mb and a translocation and inversion of 0.13 Mb, respectively.

Previous analysis based on the use of CGH revealed dramatic variation in the genomic content of eight *Thiomonas* strains (CB1, CB2, CB3, CB6, 3As, *Tm*. *arsenivorans*, Ynys1 and *Tm*. *perometabolis*) despite their phylogenetic proximity. Indeed, the 16S rDNA/*rpoA*-based phylogeny of the CB1, CB2, CB3, CB6, and 3As isolates (which share a 97% nucleotide identity), as well as DNA-DNA hybridization experiments, suggested that while they are indeed 5 different strains, they are of the same species. However, subtle physiological differences were observed among the isolates, and only 74.7% of the genome of strain 3As was sufficiently conserved to allow hybridization using the CGH approach [16]. Nevertheless, this did not allow for the comparison of genomic synteny or to detect if the genomes shared similar architectures. Here, due to the high quality of sequence assembly, the architecture of the 3As, K12 and CB2 genomes could be compared and the genomic islands identified. Dot plots were constructed for pairwise genomic comparisons among the three isolates, revealing striking similarities between 3As and K12 compared to the CB2 genome (Fig 2A). The 3As and K12 genomes are highly syntenic and similar in gene content (Fig 2A), although 3As harbors several insertions (indicated by breaks in the line) and one inversion compared to K12. In contrast, genome organization between CB2 and 3As is not well conserved due to a variety of rearrangements and inversions (Fig 2A). Since the 3As and K12 strains are phylogenetically distant but harbor similar genomic organization (Figs 1 and 2A), we hypothesize that these genomes represent the ancestral state, while that of CB2 is more derived (Fig 2B). Accordingly, three major genomic rearrangements and inversions could have occurred in the CB2 lineage after its divergence from the two others strains: (i) the translocation of a 0.1 Mb region; (ii) the inversion of a 1.8 Mb region, and (iii) the translocation and inversion of a 0.13 Mb region (Fig 2B).

The CB2 genome is larger than that of 3As and K12 due to the presence of sizable regions that may correspond to GEIs. Careful analyses were completed on the CB2, 3As and K12 genomes to define GEIs (also referred to as Regions of Genomic Plasticity or RGPs by “RGP finder”), a module in the MaGe platform (https://www.genoscope.cns.fr/agc/microscope/home/index.php). According to the criteria used, the 3As genome harbored 30 GEIs, among which 19 were identified previously using the CGH approach [16]. Twelve GEIs were specific to 3As and 5 were shared with K12. We identified 24 GEIs in the K12 genome, 11 of which were specific to this strain. We then carefully analyzed the 20 GEIs identified in CB2, which were > 5 kb, and accounted for more than 27.8% (1, 065 CDS) of the genome (S2 and S3 Tables). The island identified as RGP19 was composed of at least two distinct islands, labeled RGP19 and RGP20 (S3 Table), since these genes were found in a different scaffold. Remarkably, genes involved in plasmid conjugation (*tra* or *trb* genes) were found on five of these GEIs, suggesting that these sequences were perhaps originally acquired by plasmid conjugation and integration. Four CB2 GEIs, i.e. RGP5 (29.8 kb, 26 genes), RGP8 (35.7 kb, 32 genes), RGP12 (36.3 kb, 33 genes) and RGP17 (9.9 Kb, 12 genes) contain genes with homologs in distantly related bacteria (https://www.genoscope.cns.fr/agc/microscope/home/index.php) but not in 3As and K12, or in other *Thiomonas* genomes (S3 Table). In RGP12, of the 33 genes 4 genes were found duplicated in CB2 in another GEI and involved in urea transport. Overall, these data suggests that CB2 acquired the corresponding genes via horizontal gene transfer. The 3As and K12 genomes shared five and four distinct GEIs with CB2, respectively, while only two islands (RGP1 and 13) were found in all three genomes. Interestingly, genes found in RGP7 and a large part of RGP19 of CB2 were present in all other AMD strains (CB1, CB3, CB6, 3As, ACO3, and ACO7) but not in K12, suggesting that these two islands were most likely important for survival in AMD. The RGP7 contains almost exclusively proteins of unknown function, while RGP19 carries genes involved in As resistance and transformation (S3 Table) and was further analyzed in detail.

### Detailed analysis of the *ars* and *aio* genes of CB2, involved in As metabolism

Microbial mechanisms to mitigate the toxic effects of As include the expression of *ars* genes, which encode an arsenite (As(III)) efflux pump (*arsB*) and an arsenate (As(V)) reductase (*arsC*) and allow bacteria to reduce arsenate to arsenite in order to ultimately extrude it from the cell [35]. These genes are usually present as a three-gene operon, *arsRBC*, with *arsR* encoding a transcriptional regulator, or an extended five-gene operon *arsRDABC*, with *arsA* encoding an ATPase involved in As(III) extrusion, and *arsD* encoding an arsenite chaperone that transfers arsenite from glutathione-bound complexes to the ArsA subunit of the ArsAB complex. The *arsA* and *arsD* genes supposedly confer higher levels of resistance [35]. Another mechanism of As resistance involves the *aio* operon that encodes proteins involved in detoxifying arsenite by oxidizing As(III) to arsenate As(V) or utilizing As(III) as an electron donor [35].

**Fig 3:**
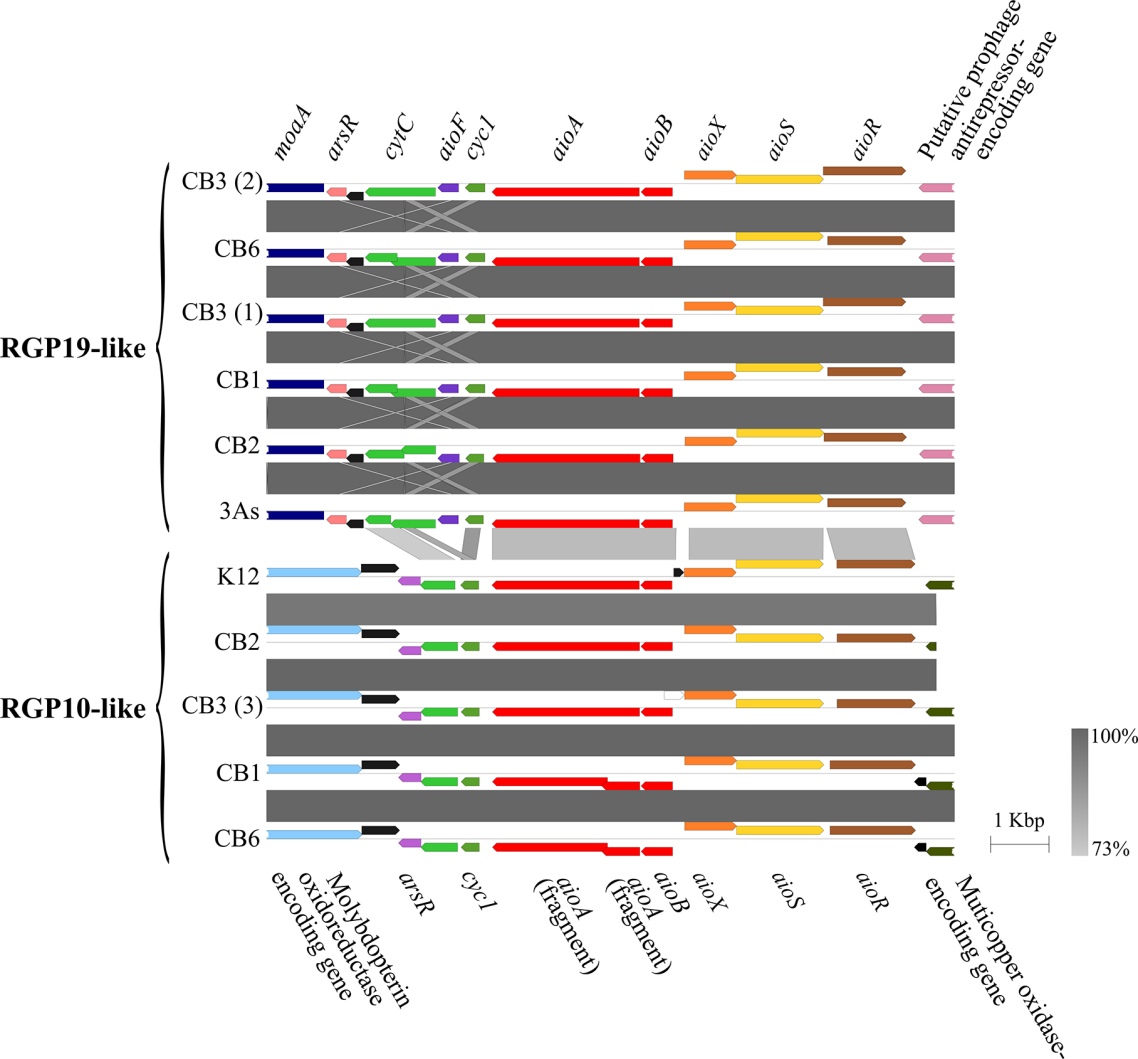
Synteny of the *aio* loci of the *Thiomonas* spp. isolates. The top portion represents the RGP19-like region present in each genomes and the bottom portion represents the RGP10-like region. The % of nucleotide identity is expressed along a grey scale. The genes involved in the arsenic oxidation are localized on the *aio* operon (red). The *aioX* (orange), *aioR* (brown) and *aioS* (yellow) code for proteins involved in regulation and expression of *aioBA*. The cytochrome genes (green) are likely involved in electron transfer. The *moaA* gene (dark blue) is involved in the biosynthesis of molybdopterin, an essential co-factor for the catalytic activity of the protein. Black arrow represents CDS with no known function. Figures were generated with Easyfig [50].

**Fig 4:**
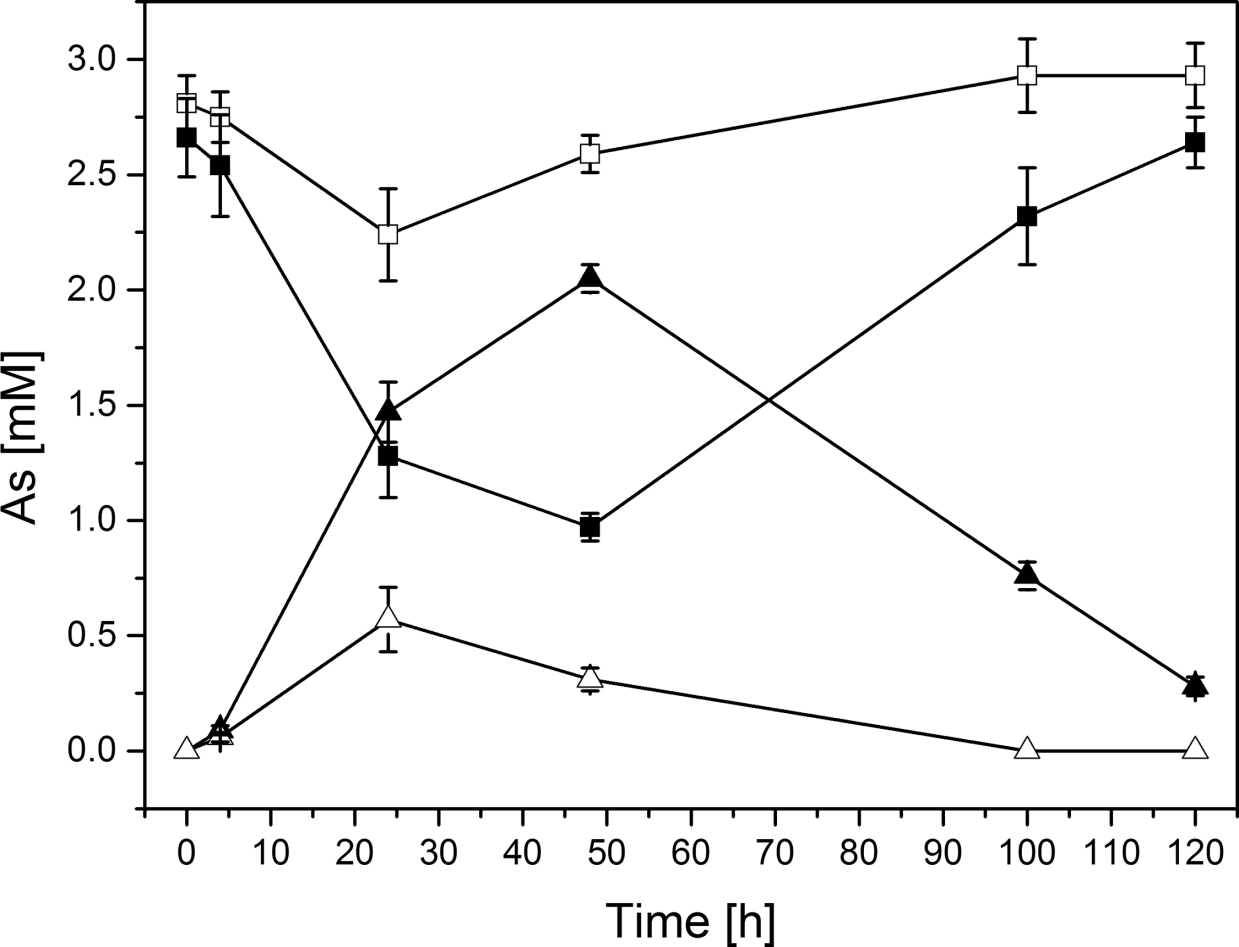
CB2 capacity to oxidize arsenite. Concentrations of As(III) (squares) and As(V) (triangles) are shown for CB2 cells grown in m126 medium in the presence of 2.66 mM of As(III) (full symbols) and m126 medium in the presence of 2.66 mM of As(III) and supplemented with glucose (hollow symbols). Error bars indicate standard deviations of triplicate cultures. No As(III) oxidation was observed in abiotic controls (data not shown).

The CB2, K12, and 3As *Thiomonas* genomes harbor an *arsRICB* operon, sharing more than 97% of similarity at the nucleotide level, that is not located in a GEI but in a very conserved region of these three genomes. Both CB2 and 3As have an additional *arsRDACB* operon with a shared 91% identity at the nucleotide level, located in a GEI in both genomes. In CB2, this operon is found on RGP19, directly upstream from an *aio* locus, while in 3As, it is located on The ThGEI-O described previously [16]. In CB2, two versions of the *aio* operon, sharing less than 90% identity at the nucleotide level, are located in two distinct GEIs (referred to from now on as “arsenic genomic islands”) (Fig 3). One copy is encoded in RGP19 of CB2, while the second is located in RGP10. In contrast, a single *aio* operon is found in 3As [16] and in K12 (this work). The CB2 AioA and AioB proteins encoded by the operon located on RGP19 both share 100% identity with the proteins encoded by 3As, and 84% and 88% identity with those of K12, respectively (Table 1). However, the AioA and AioB encoded by the operon on RGP10 share 82% and 89% identity with the 3As proteins and 87% and 98% identity with those of K12 (Fig 3 and Table 1). These observations revealed than one copy of the *aio* locus and associated genes of CB2 (located on RGP19), were more related to those in 3As, whereas the second copy of the *aio* and associated genes in CB2 (located on RGP10), were more related to those found in the unique *aio* locus of K12 (Fig 3, Table 1 and S2 Fig). To test if the genes are functional in CB2, we assessed the extent of As(III) oxidation, and found that more than 60% of As(III) was oxidized to As(V) within 48 h, followed by a slow reduction of As(V) during the stationary growth phase (Fig 4). In other *Thiomonas* isolates, where arsenite oxidation is involved in energy production, this activity was reduced in the presence of a preferred substrate. In our work, the addition of glucose to m126 caused a severe inhibition of As(III) oxidation in CB2 (Fig 4), suggesting that CB2 may use either arsenite or glucose as an electron donor and the inhibition of the arsenite oxidation could be due to a catabolite repression. Finally, we investigated if both copies of the *aio* genes were expressed in two growth conditions. First, RNAseq was performed on cells grown in conditions favoring biofilm formation (without shaking) and revealed that the expression of the *ars* and the *aio* genes (two copies of each operon), and also other genes from these two islands, are induced by As(III) (S4 Table). Second, a Western blot was performed to determine if both copies were synthetized during aerobic growth. In shaken liquid cultures, we observed that indeed, the two *aioA* genes were expressed only in the presence of As(III) (S3 Fig), as was found previously in 3As [18]. Altogether, these experiments demonstrated that both CB2 *aioAB* operons are functional.

**Table 1:** Percent of identity between AioA and AioB of CB2, 3As and K12. Whereas both 3As and K12 encoded a single copy of AioA and AioB, CB2 encoded two copies of each protein, one in RGP19 and another in RGP10. AioA and AioB of CB2 encoded in the RGP19 shared more identity with AioA and AioB of 3As (99.9% and 100% respectively). However, AioA and AioB encoded on the RGP10 share more identity with AioA and AioB of K12 (86.9% and 98.3% respectively).

	AioA				AioB			
	3As	K12	CB2-1	CB2-2	3As	K12	CB2-1	CB2-2
3As	-	83.7	99.9	82.2	-	87.7	100	88.8
K12	83.7	-	83.8	86.9	87.7	-	87.7	98.3
CB2-1	99.9	83.8	-	82.4	100	87.7	-	88.8
CB2-2	82.2	86.9	82.4	-	88.8	98.3	88.8	-

### Comparison of the two CB2 arsenic genomic islands with those found in other *Thiomonas* strains

**Fig 5:**
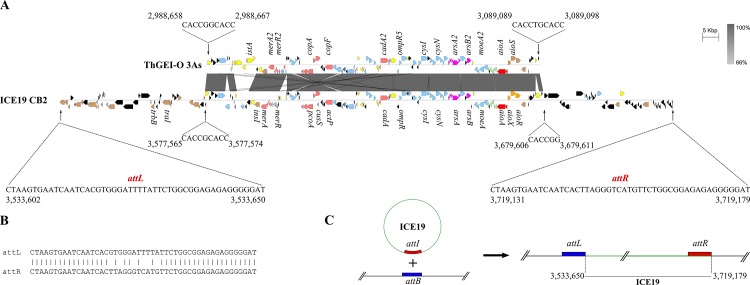
Schematic diagram of ICE19 in CB2. This figure shows one part of the arsenic island of CB2 (RGP19) that has the characteristics of an ICE (called ICE19) (A) Alignment of ICE19 of *Thiomonas* sp. CB2 with its cognate portion found in the strain 3As (ThGEI-O). The arsenic island RGP19 of CB2 is localized in a different genomic region that ThGEI-O in 3As. The ThGEI-O of 3As (upper portion of the figure) is localized in a gene encoding a 4Fe-4S ferredoxin which is intact in CB2. Direct repeat sequences are indicated. (B) Sequence comparison of the almost perfect direct repeat flanking the ICE19. *attL*: left DR; *attR*: right DR. (C) Schematic representation of the integrated and circular form of the ICE19 of CB2, which were detected by PCR. The sequence *attI* is identical to *attR* and *attB* identical to *attL*. The % of nucleotide identity is expressed along a grey scale. Figures were generated with Easyfig [50].

RGP19 is 338.7 kb long and contains genes implicated in resistance to various heavy metals (As, mercury, copper, zinc, and cadmium). Genes involved in sulfate assimilation (*cys* genes) and biotin synthesis (*bio* genes), were also present in this genomic island. In fact, many integrases are also located on RGP19, as well as genes involved in conjugation, including phage integrase and relaxase. 49 bp nearly perfect direct repeats are located at the left extremity of RGP19 and between gene THICB2v3-780013 and -780014, and the region between these two repeats contains *tra* and *trb* genes involved in conjugation (Fig 5). The excision and circularization of this part have been confirmed by PCR in CB2 (Fig 5C and data not shown). All these observations emphasized the fact that this region has been acquired after insertion and is or, at some point, was mobile. Consequently, this part of RGP19 (from 3533461 to 3796598, 185,5 Kb) has the characteristics of an integrative and conjugative element (ICE) [36], and has been designated ICE19 and the repeats designed *attL* and *attR* (Fig 5). We observed that the arsenic island is not syntenic in 3As as compared to CB2 and only part of this ICE19 is found in 3As (in the genomic island previously named ThGEI-O [16], Fig 5). In particular, the *tra* and *trb* genes were not found in the ThGEI-O and therefore this island has not the characteristics of an ICE. In 3As, ThGEI-O is located in the middle of a gene encoding a 4Fe-4S ferredoxin, whereas in CB2, ICE19 is found near the *fbp* gene encoding fructose-1,6-bisphosphatase and the Thr tRNA. The presence of direct repeats (DR) at the both sides of the ThGEI-O (RGP19-like island) in 3As was previously described [16] and indicates a probable insertion by site-specific recombination in the gene encoding a 4Fe-4S ferredoxin. Interestingly, similar repeats are also found in the ICE19 of CB2, although one is slightly different, perhaps due to punctual mutations occurred after the insertion or rearrangement of genes in this region (Fig 5). These observations suggest that the two islands, ThGEI-O in 3As and RGP19 in CB2, may have similar origin but evolved differently in the two strains.

RGP10 is shorter (91.2 kb) than RGP19 and contains genes encoding transporters as well as those involved in resistance to cobalt or acriflavine. In addition, RGP10 encodes a hydrogenase similar to that of a gastropod endosymbiont Gammaproteobacteria, which possibly acquired the relevant genes by HGT [37]. A region containing genes similar to those in RGP10 was detected in K12, however, it is shorter in K12 in comparison to RGP10 (S2 Fig). In CB2, an additional region was identified that is not harbored by K12, which includes genes encoding transposases, integrases, and proteins of unknown function. All in all, these observations suggest that the two CB2 arsenic islands RGP19 and RGP10 are different, at least in part, from those of 3As and K12, respectively.

To further analyze these two arsenic genomic islands, we searched for the genes involved in As metabolism in the CB1, CB3, CB6, ACO3, and ACO7 draft genomes. Interestingly, multiple copies of the *aio/ars* genes were in some strains (Fig 3). In fact, the ACO3 and ACO7 genomes are very similar to the 3As genome and encode only one copy of the *aio* genes (https://www.genoscope.cns.fr/agc/microscope/home/index.php). CB1 and CB6 strains harbour one copy of the *aio* genes, found in RGP10, with a frameshift in the *aioA* sequence, and one copy of the *aio/ars* genes is found in CB2 RGP19 (Fig 3 and S2 Fig). A Western blot revealed that only one copy is indeed expressed in CB1, CB6, ACO3, and ACO7 strains with a similar apparent molecular weight when grown in the presence of arsenite (S3C Fig). Interestingly, the genome of the CB3 strain contains five copies of the *ars* genes as well as four copies of the *aio* loci, which were clearly located on different contigs. The *aio* genes in CB2 RGP19 were identified in 3 copies in CB3 (Fig 3); however, one *aio* cluster was only partially intact (only one *aioA* fragment and no *aioB*) and found on a short scaffold. Interestingly, at least two copies of *aioA* were expressed in the presence of arsenite in CB3 and the proteins migrated similarly to the two CB2 AioA proteins (S3C Fig). The synteny around the *aio* genes was analysed in more detail in these *Thiomonas* strains (Fig 3 and S2 Fig). It has to be mentioned that this synteny analysis was limited by the scaffolds limits in each of the draft genomes. Indeed, the synteny of the entire CB2 RGP was occasionally not clarified in other genomes since the scaffold organization could not be deciphered. Nevertheless, this analysis revealed that in all strains, the synteny of the *aio*/*ars* genes was conserved (Fig 3), whereas the synteny of the other genes from these islands was not (S2 Fig). These observations further support the hypothesis that these two arsenic islands have evolved differently in these *Thiomonas* strains through multiple genomic rearrangements or HGT.

Finally, we investigated if the number of *aio* and *ars* genes influenced resistance to As(III) and its oxidation efficiency in *Thiomonas* strains. The MIC for As(III) was previously determined in liquid culture, however, in this study we tested the As(III) resistance on solid media with a narrow range of As(III) concentrations. We observed that the CB2 and CB3 strains are slightly more resistant than K12, ACO3, and ACO7 while the strains 3As, CB1, and CB6 demonstrated the lowest levels of As(III) resistance (S5 Table). We also determined if the kinetics of As(III) oxidation to As(V) were similar in all these strains by measuring the As(III) and As(V) concentrations over 120 h (S4 Fig). The As(III) oxidation activity of K12 was lower compared to the strains isolated from the AMD strains (CB1, CB2, CB3, CB6 and 3As). No dramatic difference in the As(III) oxidation was observed between the AMD strains suggesting that despite several copies of *aio* genes, CB2 and CB3 did not oxidize As(III) more rapidly than the other strains, this activity was even slightly slower in CB2. In contrast, once the As(III) was completely oxidized, As(V) was then reduced in 3As, CB1, and CB6. Such As(V) reduction activity was not observed in CB2 or CB3 in 1.3 mM As(III). These results suggest that arsenate reduction is either less efficient or that As(III) oxidation activity is maintained for a longer time period in these two strains. Altogether, these data revealed that in relation to the genes involved in As metabolism, the As(III) resistance and oxidation capacities are different between these closely related strains.

## Discussion

With the growing number of genome sequences available, it is now possible to utilize comparative tools to understand adaptations that microorganisms have acquired in diverse environments. Here, we compared the CB2 genome to that in other *Thiomonas* strains, including two complete (3As and K12) and five draft (ACO3, ACO7, CB1, CB3 and CB6) genomes. These isolates, as well as CB2 and 3As, were isolated from an AMD rich in toxic metals ([14,16] and this work), while K12 was originally obtained from a sewer in Hamburg, an environment not known for high As concentrations [17]. The genetic relatedness of K12 with other *Thiomonas* strains has never been analyzed previously. Here, a detailed phylogenetic analysis was completed that demonstrated that 3As, CB1, CB6, ACO3, and ACO7 are very closely related and clade with CB3 and CB2, while the K12 strain clearly diverges from this group.

CB1, CB6, 3As, ACO3, and ACO7 expressed only one copy of the *aio* genes, while CB2 and CB3 have two or more copies which are expressed, each likely playing a role in As tolerance and metabolism. To date, the Carnoulès AMD, which is highly contaminated with As(III), is the only location that has yielded isolates with multiple copies of the *aio* operon. Expressing two *aio* operons may allow CB2 and CB3 strains to maintain arsenite oxidation activity for a longer time period, as suggested by the results we’ve obtained in this study, ultimately allowing these strains to be slightly more resistant to As(III) than the others assessed.

Due to a robust assembly, the comparison among K12, CB2, and 3As allowed us to define essential GEIs, which might be important for survival in AMD. In CB2, we described 20 GEIs, while 30 were found in 3As ([16] and this work). Genes found in only two GEIs were shared between all the isolates from AMD strains but not in K12, revealing that there is not a large pool of AMD-specific genes in *Thiomonas* strains from the Carnoulès AMD. In fact, our data demonstrated that the *Thiomonas* genomes are variable, due to the presence, expansion (possibly via gene duplication and/or HGT), or absence of several GEIs. In addition, several *Thiomonas* strains have at least one plasmid. Remarkably, the size and content of the plasmids found in the strains analyzed here are variable and the functions they encoded were different and may be specific to some strains. Thus, a large portion of the genome in these strains may confer different ecological capacities and functions, acting as a flexible region influencing gene content diversity and suggesting that they adapt differently to their niches. Finally, the K12 genome (3, 467 genes) is smaller than the others (which range from 3, 628 to 4, 508 genes) possibly indicating its native environment requires fewer genes for survival. Additionally, we found a larger number of CDS with unknown functions in the AMD strains, in particular in RGP7, which is found in all the strains isolated from AMD. It may be possible that these genes confer specific capacities not yet characterized, which could allow for survival in AMD.

Our comparative analyses revealed that the CB2 chromosome has a unique architecture in comparison to 3As and K12. Surprisingly, the 3As genome structure was more similar to K12, possibly indicating that the CB2 genome underwent specific genomic events, exemplified by three major inversions and rearrangements compared to 3As. Furthermore, some CB2 GEIs appear to contain multiple islands, most likely acquired after the divergence of this strain. Some of these GEIs, in particular the ICE19 possibly allowing for survival in the harsh AMD environment may have been acquired by HGT [38,39]. In particular, within biofilm structures, HGT is more likely to shuffle genes among organisms [40,41]. Indeed CB2 forms biofilms, which are stimulated in the presence of arsenic [30], which may have played a role in the acquisition of exogenous DNA. Previous work demonstrated genetic exchange is more common between closely related organisms [42], thus *Thiomonas* sp. could potentially transfer the portions of the GEIs that confer a selective survival advantage to other *Thiomonas* strains. Our data and a recent analysis of GEIs involved in As metabolism [43] suggest that both vertical inheritance and HGT favor the spread of such *aio/ars* genes. Thus, rearrangement, HGT and gene loss events probably impacted CB2 genome content after strain divergence, leading to the formation of similar but not identical composite GEIs. In particular, the arsenic islands may have been modified by HGT or rearrangement processes, since it appears that the gene content of these islands is similar but not identical in *Thiomonas* strains.

The analysis of the *Thiomonas* genomes is a step towards understanding environmental pressures on genome evolution. The high frequency of modifications within these sequences possibly depicts the first phase in the creation of novel species and the extinction of others [5,44,45]. Readily shuffling DNA allows microbial community members to sample the environment for potentially beneficial genes, which has implications in both community diversity and strain-specific lineage survival [39,41]. Our results clearly demonstrate the importance of genomic islands in *Thiomonas* adaptation. These islands allow organisms to inhabit diverse environments by providing genomic plasticity and the rapid acquisition of functional traits. In the case of *Thiomonas*, the environment appears to have played an important role in driving the acquisition of genomic islands, and is possibly the source of selective pressures leading to rearrangements and HGT. Therefore, in order to obtain a detailed understanding of bacterial adaptation, speciation, and evolution, as well as to delineate lineages, a holistic view taking into account bacterial species as well as traditional metrics needs to be adopted.

## Supporting Information

S1 Fig Plasmid detection and visualization in CB1, CB2, CB3, CB6, 3As, and K12 *Thiomonas* strains in agarose gels after ethidium bromide staining. K3: the 3 plasmids from *Klebsiella pneumoniae* with estimated sizes of 225 kb, 130 kb and 45 kb. One extra chromosomal element less than 40 kb was detected in CB2, CB3, and K12, while an additional one of approximately 40 kb was detected in 3As, CB1, CB3, and K12.
(TIF)

S2 Fig Comparison of the *Thiomonas* genes similar to those found in RGP19 and RGP10 in CB2 genome. The synteny between the 3As, K12, and CB2 genomes and CB1, CB3, and CB6 scaffolds is represented. The % of nucleotide identity is expressed on a grey scale. Genes: red = *aio* genes; purple = *ars* genes; pink: genes involved in metals resistance (Cu, Hg, Cd…); yellow: genes encoding transposases, integrases; brown: mobile genetic elements associated genes (including *mob*, *tra*, and *trb)*; orange and green: genes conserved around *aioBA* (see Fig 3); black: unknown function and /or no homology known; blue: others genes. Figures were generated with Easyfig (Sullivan *et al*., 2011). *Thiomonas sp*. CB2 has two *aioBA* operons localized on the RGP19 and RGP10. The strains 3As and K12 have one copy localized in a region of conserved synteny with the RGP19 (RGP19-like) and the RGP10 (RGP10-like) respectively. *Thiomonas* sp. CB3 have two *aioBA* localized in a RGP19-like region and another found in a RGP10-like region. Both CB1 and CB6 have two *aioBA* copies in a RGP19-like and RGP10-like region respectively, but with a frameshift in *aioA* in the latter region.
(TIF)

S3 Fig Western blots to detect *aioBA* operon expression in *Thiomonas* strains. Expression of two AioA proteins in the presence of arsenic is indicated with arrows. (A) Results from a planktonic culture of CB2 grown without or with arsenic. The two AioA are induced in the presence of As(III). (B) Planktonic cultures of CB2 grown at a range of concentration of As (III). The two AioA are expressed from 0.67 mM to 2.66 mM of As(III). (C) Results from planktonic cultures of CB1, CB2, CB3, CB6 and 3As grown with 1.33 mM of As(III). Two AioA are expressed for CB2 and CB3 and only one of similar weight for CB1, CB6, ACO3 and ACO7.
(TIF)

S4 Fig Capacity of *Thiomonas* strains to oxidize arsenite, As(III), to arsenate, As(V). Concentrations of As(III) (squares) and As(V) (triangles) are shown for cells grown in m126 with an initial concentration of 1.33 mM As(III). On the left of the figure the concentrations measured at t_0h_, t_+24h_, t_+48h_, t_+72h_ and t_+120h_ are represented, while on the right the concentrations measured at t_0h_, t_+6h_, t_+12h_ and t_+24h_ are shown. Error bars indicate standard deviation of data obtained from triplicate cultures. The As(III) oxidation activity of K12 was lower than the strains from the AMD. After 24 h, and once all As(III) was oxidized, 3As, CB1, and CB6 reduced the As(V), which was not observed for CB2 or CB3.
(TIF)

S1 Table General characteristics of the *Thiomonas* genomes. Calculation of repetitive regions did not include undetermined (‘N’) bases. The genome size and gene content range from 3.4 Mb and 4, 367 CDS (K12) to 4.3 Mb and 4, 508 CDS (CB3). *Thiomonas* sp. CB3 also has a higher proportion of repetitive regions (19.21%). The others general features are similar between the strains.
(DOCX)

S2 Table Regions of genomic plasticity (RGP) identified after comparison of CB2 with 3As and K12. The RGP were identified with the *RGP finder* module in MaGe, which identify synteny breaks and search for HGT features (tRNA hotspots, genes involed in mobility), and for compositional bias (AlienHunter (Vernikos and Parkhill, 2006), SIGI-HMM (Waack *et al*., 2006), and GC deviation computation).
(DOCX)

S3 Table Details of the RGP found in CB2 genome. (XLSX)

S4 Table RNAseq results for genes that are significantly more expressed in the presence than in the absence of arsenite. The *ars* and the *aio* genes (two copies of each operon), as well as other genes from these two islands (RGP10 and RGP19) are induced by As(III).
(XLSX)

S5 Table Minimal inhibitory concentration (MIC) of As(III) for *Thiomonas* strains on solid m126. *Thiomonas* spp. CB2 and CB3 are slightly more resistant than the others strains. *Thiomonas* spp. 3As, CB1 and CB6 demonstrate lower levels of resistance to As(III).
(DOCX)

## References

[1] Coleman ML, Sullivan MB, Martiny AC, Steglich C, Barry K, Delong EF, et al Genomic islands and the ecology and evolution of *Prochlorococcus* . Science. 2006;311: 1768–1770. 10.1126/science.1122050 16556843

[2] Juhas M, van der Meer JR, Gaillard M, Harding RM, Hood DW, Crook DW. Genomic islands: tools of bacterial horizontal gene transfer and evolution. FEMS Microbiol Rev. 2009;33: 376–393. 10.1111/j.1574-6976.2008.00136.x 19178566 PMC2704930

[3] Penn K, Jenkins C, Nett M, Udwary DW, Gontang EA, McGlinchey RP, et al Genomic islands link secondary metabolism to functional adaptation in marine *Actinobacteria* . ISME J. 2009;3: 1193–1203. 10.1038/ismej.2009.58 19474814 PMC2749086

[4] Cohan FM. What are bacterial species? Annu Rev Microbiol. 2002;56: 457–487. 10.1146/annurev.micro.56.012302.160634 12142474

[5] Cohan FM, Perry EB. A systematics for discovering the fundamental units of bacterial diversity. Curr Biol CB. 2007;17: R373–386. 10.1016/j.cub.2007.03.032 17502094

[6] Koeppel A, Perry EB, Sikorski J, Krizanc D, Warner A, Ward DM, et al Identifying the fundamental units of bacterial diversity: a paradigm shift to incorporate ecology into bacterial systematics. Proc Natl Acad Sci U S A. 2008;105: 2504–2509. 10.1073/pnas.0712205105 18272490 PMC2268166

[7] Cheng H, Hu Y, Luo J, Xu B, Zhao J. Geochemical processes controlling fate and transport of arsenic in acid mine drainage (AMD) and natural systems. J Hazard Mater. 2009;165: 13–26. 10.1016/j.jhazmat.2008.10.070 19070955

[8] Casiot C, Morin G, Juillot F, Bruneel O, Personné JC, Leblanc M, et al Bacterial immobilization and oxidation of arsenic in acid mine drainage (Carnoulès creek, France). Water Res. 2003;37: 2929–2936. 10.1016/S0043-1354(03)00080-0 12767295

[9] Bruneel, Duran R, Casiot C, Elbaz-Poulichet F, Personné J-C. Diversity of microorganisms in Fe-As-rich acid mine drainage waters of Carnoulès, France. Appl Environ Microbiol. 2006;72: 551–556. 10.1128/AEM.72.1.551-556.2006 16391091 PMC1352176

[10] Volant A, Desoeuvre A, Casiot C, Lauga B, Delpoux S, Morin G, et al Archaeal diversity: temporal variation in the arsenic-rich creek sediments of Carnoulès Mine, France. Extrem Life Extreme Cond. 2012;16: 645–657. 10.1007/s00792-012-0466-8 22714283

[11] Volant A, Bruneel O, Desoeuvre A, Héry M, Casiot C, Bru N, et al Diversity and spatiotemporal dynamics of bacterial communities: physicochemical and other drivers along an acid mine drainage. FEMS Microbiol Ecol. 2014;90: 247–263. 10.1111/1574-6941.12394 25070063

[12] Bruneel, Personné J-C, Casiot C, Leblanc M, Elbaz-Poulichet F, Mahler BJ, et al Mediation of arsenic oxidation by *Thiomonas* sp. in acid-mine drainage (Carnoulès, France). J Appl Microbiol. 2003;95: 492–499. 12911697 10.1046/j.1365-2672.2003.02004.x

[13] Battaglia-Brunet F, Joulian C, Garrido F, Dictor M-C, Morin D, Coupland K, et al Oxidation of arsenite by *Thiomonas* strains and characterization of *Thiomonas arsenivorans* sp. nov. Antonie Van Leeuwenhoek. 2006;89: 99–108. 10.1007/s10482-005-9013-2 16341463

[14] Duquesne K, Lieutaud A, Ratouchniak J, Muller D, Lett M-C, Bonnefoy V. Arsenite oxidation by a chemoautotrophic moderately acidophilic *Thiomonas* sp.: from the strain isolation to the gene study. Environ Microbiol. 2008;10: 228–237. 10.1111/j.1462-2920.2007.01447.x 17894815

[15] Bryan, Marchal M, Battaglia-Brunet F, Kugler V, Lemaitre-Guillier C, Lièvremont D, et al Carbon and arsenic metabolism in *Thiomonas* strains: differences revealed diverse adaptation processes. BMC Microbiol. 2009;9: 127 10.1186/1471-2180-9-127 19549320 PMC2720973

[16] Arsène-Ploetze F, Koechler S, Marchal M, Coppée J-Y, Chandler M, Bonnefoy V, et al Structure, function, and evolution of the *Thiomonas* spp. genome. PLoS Genet. 2010;6: e1000859 10.1371/journal.pgen.1000859 20195515 PMC2829063

[17] Milde K, Sand W, Wolff W, Bock E. *Thiobacilli* of the corroded concrete walls of the Hamburg sewer system. J Gen Microbiol. 1983;129: 1327–1333.

[18] Slyemi D, Moinier D, Brochier-Armanet C, Bonnefoy V, Johnson DB. Characteristics of a phylogenetically ambiguous, arsenic-oxidizing *Thiomonas* sp., *Thiomonas arsenitoxydans* strain 3As(T) sp. nov. Arch Microbiol. 2011;193: 439–449. 10.1007/s00203-011-0684-y 21409355

[19] Moreira D, Amils R. Phylogeny of *Thiobacillus cuprinus* and other mixotrophic thiobacilli: proposal for *Thiomonas* gen. nov. Int J Syst Bacteriol. 1997;47: 522–528. 9103643 10.1099/00207713-47-2-522

[20] Bertin, Heinrich-Salmeron A, Pelletier E, Goulhen-Chollet F, Arsène-Ploetze F, Gallien S, et al Metabolic diversity among main microorganisms inside an arsenic-rich ecosystem revealed by meta- and proteo-genomics. ISME J. 2011;5: 1735–47. 10.1038/ismej.2011.51 21562598 PMC3197163

[21] Rinkel M, Hubert JC, Roux B, Lett MC. Identification of a new transposon Tn5403 in a *Klebsiella pneumoniae* strain isolated from a polluted aquatic environment. Curr Microbiol. 1994;29: 249–254. 7765419 10.1007/BF01577436

[22] Laemmli UK. Cleavage of structural proteins during the assembly of the head of bacteriophage T4. Nature. 1970;227: 680–685. 5432063 10.1038/227680a0

[23] Vallenet D, Labarre L, Rouy Z, Barbe V, Bocs S, Cruveiller S, et al MaGe: a microbial genome annotation system supported by synteny results. Nucleic Acids Res. 2006;34: 53–65. 10.1093/nar/gkj406 16407324 PMC1326237

[24] Vallenet D, Engelen S, Mornico D, Cruveiller S, Fleury L, Lajus A, et al MicroScope: a platform for microbial genome annotation and comparative genomics. Database J Biol Databases Curation. 2009;2009: bap021. 10.1093/database/bap021 PMC2790312 20157493

[25] Vallenet D, Belda E, Calteau A, Cruveiller S, Engelen S, Lajus A, et al MicroScope—an integrated microbial resource for the curation and comparative analysis of genomic and metabolic data. Nucleic Acids Res. 2013;41: D636–647. 10.1093/nar/gks1194 23193269 PMC3531135

[26] Boetzer M, Henkel CV, Jansen HJ, Butler D, Pirovano W. Scaffolding pre-assembled contigs using SSPACE. Bioinformatics. 2011;27: 578–579. 10.1093/bioinformatics/btq683 21149342

[27] Kurtz S, Phillippy A, Delcher AL, Smoot M, Shumway M, Antonescu C, et al Versatile and open software for comparing large genomes. Genome Biol. 2004;5: R12 10.1186/gb-2004-5-2-r12 14759262 PMC395750

[28] Wheatcroft R. Changes in the *Rhizobium meliloti* Genome and the Ability to Detect Supercoiled Plasmids During Bacteroid Development. Mol Plant Microbe Interact. 1990;3: 9 10.1094/MPMI-3-009

[29] Albiger B, Hubert JC, Lett MC. Identification of the plasmid-mobilization potential of the strain *Klebsiella pneumoniae ozenae* KIIIA isolated from a polluted aquatic environment. Plasmid. 1999;41: 30–39. 10.1006/plas.1998.1372 9887304

[30] Marchal M, Briandet R, Halter D, Koechler S, Dubow MS, Lett M-C, et al Subinhibitory arsenite concentrations lead to population dispersal in *Thiomonas* sp. PloS One. 2011;6: e23181 10.1371/journal.pone.0023181 21876737 PMC3158062

[31] R Development Core Team. R: A Language and Environment for Statistical Computing. Vienna, Austria: the R Foundation for Statistical Computing; 2011 Available: http://www.R-project.org/.

[32] Gentleman RC, Carey VJ, Bates DM, Bolstad B, Dettling M, Dudoit S, et al Bioconductor: open software development for computational biology and bioinformatics. Genome Biol. 2004;5: R80 10.1186/gb-2004-5-10-r80 15461798 PMC545600

[33] Anders S, Huber W. Differential expression analysis for sequence count data. Genome Biol. 2010;11: R106 10.1186/gb-2010-11-10-r106 20979621 PMC3218662

[34] Benjamini Y, Hochberg Y. Controlling the False Discovery Rate: A Practical and Powerful Approach to Multiple Testing. J R Stat Soc Ser B Methodol. 1995;57: 289–300.

[35] Kruger MC, Bertin PN, Heipieper HJ, Arsène-Ploetze F. Bacterial metabolism of environmental arsenic—mechanisms and biotechnological applications. Appl Microbiol Biotechnol. 2013;97: 3827–3841. 10.1007/s00253-013-4838-5 23546422

[36] Bellanger X, Payot S, Leblond-Bourget N, Guédon G. Conjugative and mobilizable genomic islands in bacteria: evolution and diversity. FEMS Microbiol Rev. 2014;38: 720–760. 10.1111/1574-6976.12058 24372381

[37] Nakagawa S, Shimamura S, Takaki Y, Suzuki Y, Murakami S-I, Watanabe T, et al Allying with armored snails: the complete genome of gammaproteobacterial endosymbiont. ISME J. 2014;8: 40–51. 10.1038/ismej.2013.131 23924784 PMC3869010

[38] Denef VJ, Mueller RS, Banfield JF. AMD biofilms: using model communities to study microbial evolution and ecological complexity in nature. ISME J. 2010;4: 599–610. 10.1038/ismej.2009.158 20164865

[39] Aminov RI. Horizontal gene exchange in environmental microbiota. Front Microbiol. 2011;2: 158 10.3389/fmicb.2011.00158 21845185 PMC3145257

[40] Harrison JJ, Ceri H, Turner RJ. Multimetal resistance and tolerance in microbial biofilms. Nat Rev Microbiol. 2007;5: 928–938. 10.1038/nrmicro1774 17940533

[41] Madsen JS, Burmølle M, Hansen LH, Sørensen SJ. The interconnection between biofilm formation and horizontal gene transfer. FEMS Immunol Med Microbiol. 2012;65: 183–195. 10.1111/j.1574-695X.2012.00960.x 22444301

[42] Ochman H, Lawrence JG, Groisman EA. Lateral gene transfer and the nature of bacterial innovation. Nature. 2000;405: 299–304. 10.1038/35012500 10830951

[43] Li H, Li M, Huang Y, Rensing C, Wang G. In silico analysis of bacterial arsenic islands reveals remarkable synteny and functional relatedness between arsenate and phosphate. Front Microbiol. 2013;4: 347 10.3389/fmicb.2013.00347 24312089 PMC3834237

[44] Cohan FM. Bacterial species and speciation. Syst Biol. 2001;50: 513–524. 12116650 10.1080/10635150118398

[45] Koeppel AF, Wu M. Species matter: the role of competition in the assembly of congeneric bacteria. ISME J. 2014;8: 531–540. 10.1038/ismej.2013.180 24132075 PMC3930319

[46] Gouy M, Guindon S, Gascuel O. SeaView version 4: A multiplatform graphical user interface for sequence alignment and phylogenetic tree building. Mol Biol Evol. 2010;27: 221–224. 10.1093/molbev/msp259 19854763

[47] Guindon S, Dufayard J-F, Lefort V, Anisimova M, Hordijk W, Gascuel O. New algorithms and methods to estimate maximum-likelihood phylogenies: assessing the performance of PhyML 3.0. Syst Biol. 2010;59: 307–321. 10.1093/sysbio/syq010 20525638

[48] Jobb G, von Haeseler A, Strimmer K. TREEFINDER: a powerful graphical analysis environment for molecular phylogenetics. BMC Evol Biol. 2004;4: 18 10.1186/1471-2148-4-18 15222900 PMC459214

[49] Ronquist F, Teslenko M, van der Mark P, Ayres DL, Darling A, Höhna S, et al MrBayes 3.2: efficient Bayesian phylogenetic inference and model choice across a large model space. Syst Biol. 2012;61: 539–542. 10.1093/sysbio/sys029 22357727 PMC3329765

[50] Sullivan MJ, Petty NK, Beatson SA. Easyfig: a genome comparison visualizer. Bioinforma Oxf Engl. 2011;27: 1009–1010. 10.1093/bioinformatics/btr039 PMC3065679 21278367

