# Supplementary figures and images for: Adaptation in Toxic Environments: Arsenic Genomic Islands in the Bacterial Genus Thiomonas
Source: PLoS One. 2015 Sep 30;10(9):e0139011. doi: 10.1371/journal.pone.0139011 (PMC4589449; doi:10.1371/journal.pone.0139011)

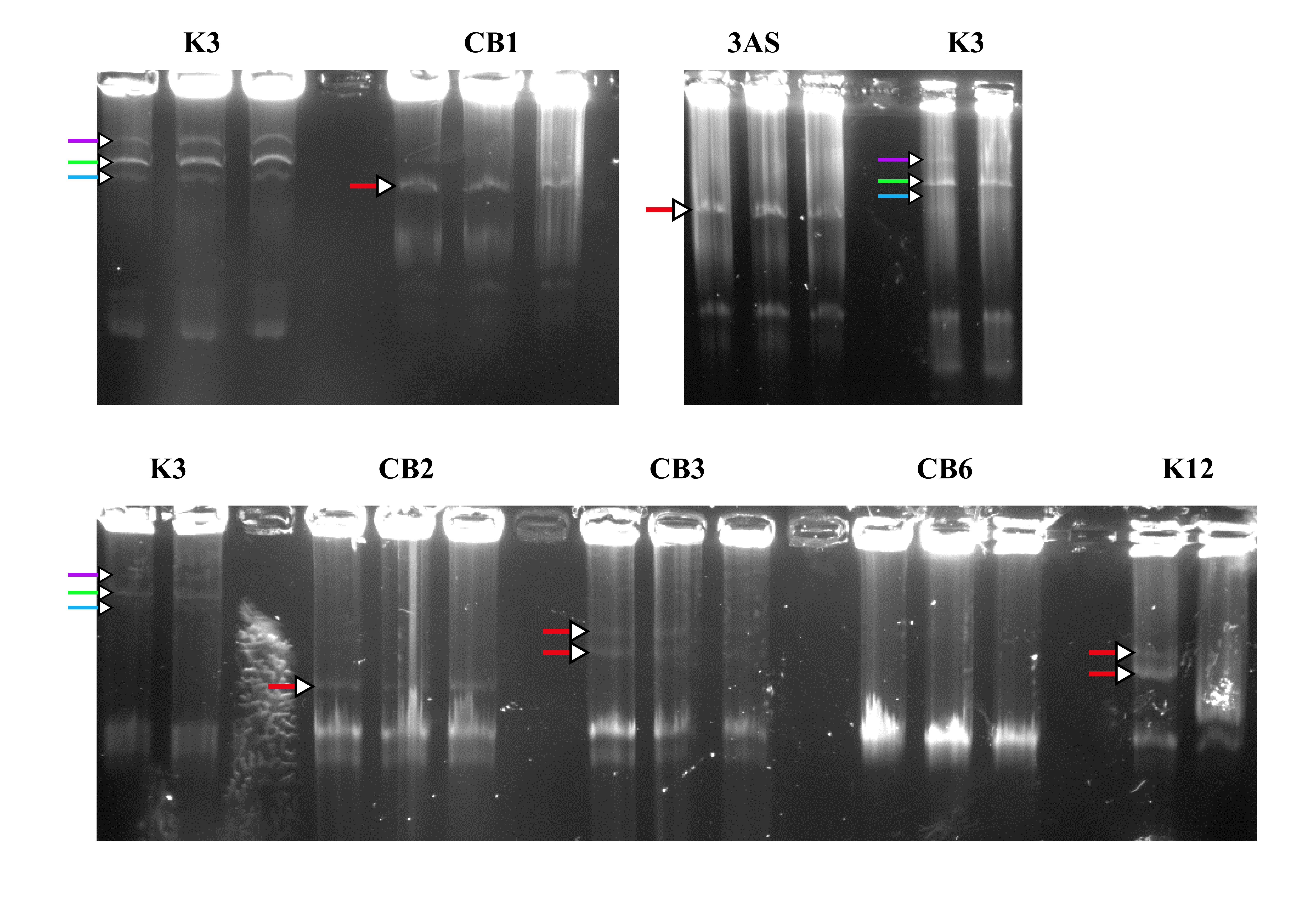

Supplement: S1 Fig — K3: the 3 plasmids from Klebsiella pneumoniae with estimated sizes of 225 kb, 130 kb and 45 kb. One extra chromosomal element less than 40 kb was detected in CB2, CB3, and K12, while an additional one of approximately 40 kb was detected in 3As, CB1, CB3, and K12. (TIF) [file pone.0139011.s001.tif]

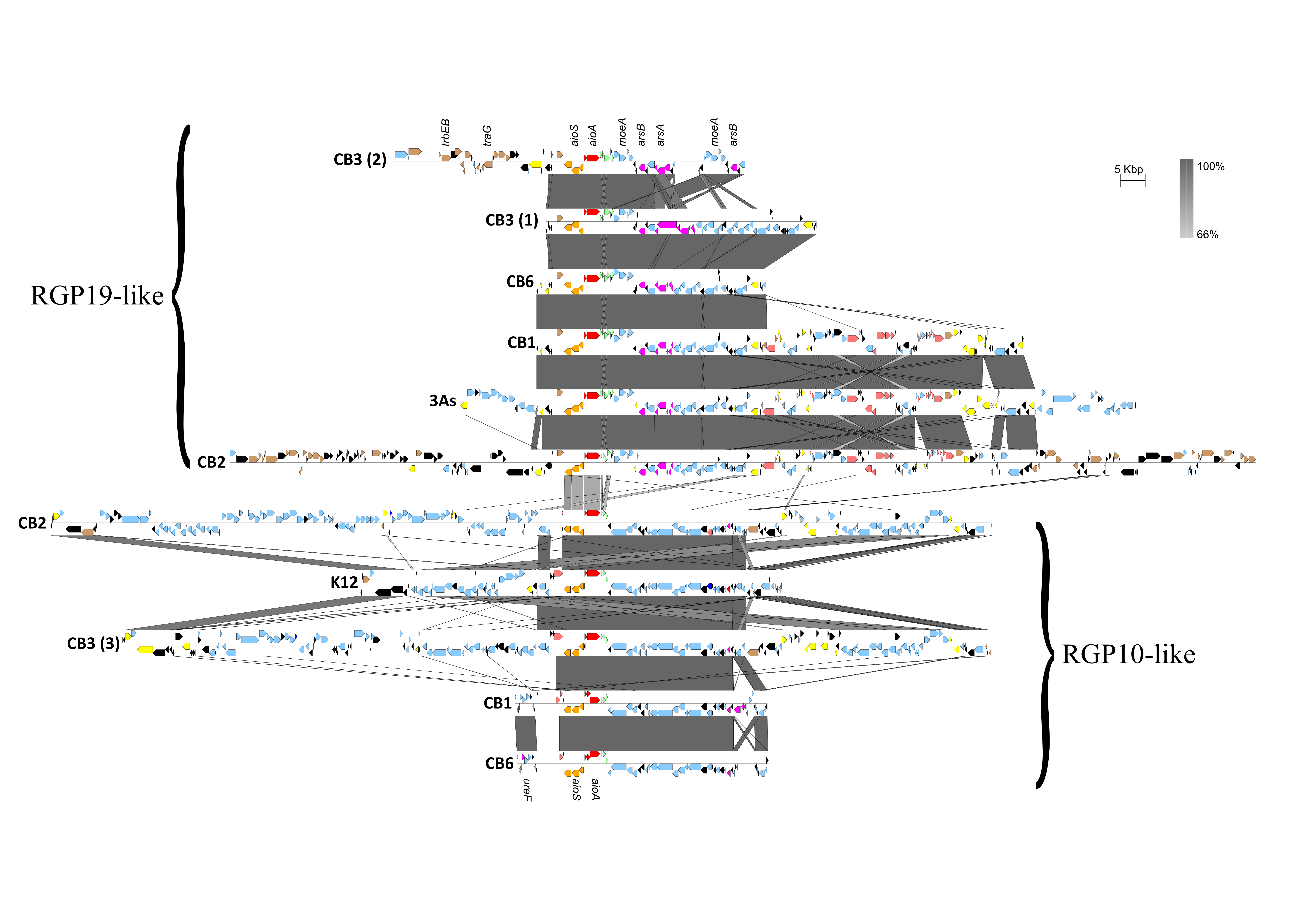

Supplement: S2 Fig — The synteny between the 3As, K12, and CB2 genomes and CB1, CB3, and CB6 scaffolds is represented. The % of nucleotide identity is expressed on a grey scale. Genes: red = aio genes; purple = ars genes; pink: genes involved in metals resistance (Cu, Hg, Cd…); yellow: genes encoding transposases, integrases; brown: mobile genetic elements associated genes (including mob, tra, and trb); orange and green: genes conserved around aioBA (see Fig 3); black: unknown function and /or no homology known; blue: others genes. Figures were generated with Easyfig (Sullivan et al., 2011). Thiomonas sp. CB2 has two aioBA operons localized on the RGP19 and RGP10. The strains 3As and K12 have one copy localized in a region of conserved synteny with the RGP19 (RGP19-like) and the RGP10 (RGP10-like) respectively. Thiomonas sp. CB3 have two aioBA localized in a RGP19-like region and another found in a RGP10-like region. Both CB1 and CB6 have two aioBA copies in a RGP19-like and RGP10-like region respectively, but with a frameshift in aioA in the latter region. (TIF) [file pone.0139011.s002.tif]

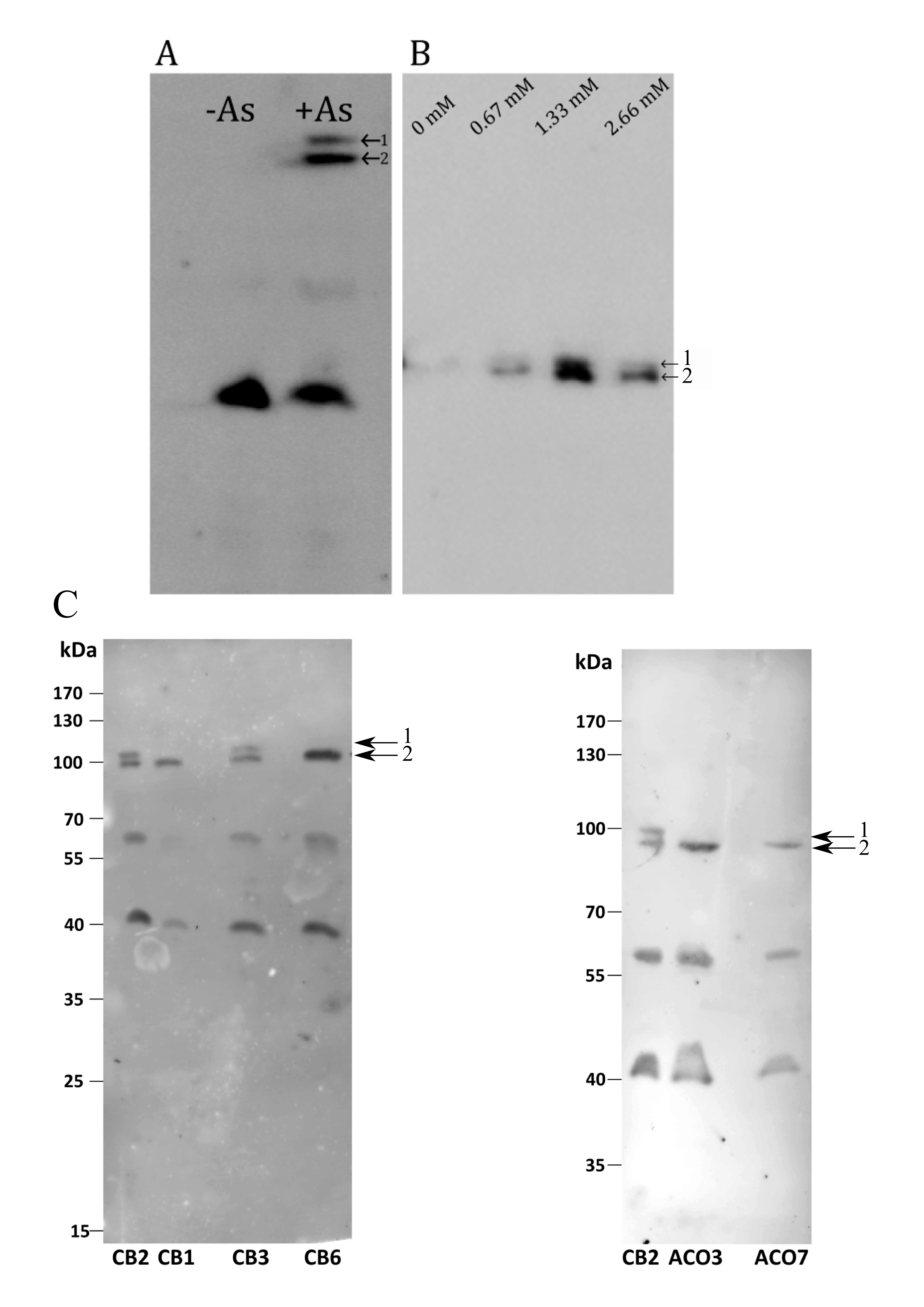

Supplement: S3 Fig — Expression of two AioA proteins in the presence of arsenic is indicated with arrows. (A) Results from a planktonic culture of CB2 grown without or with arsenic. The two AioA are induced in the presence of As(III). (B) Planktonic cultures of CB2 grown at a range of concentration of As (III). The two AioA are expressed from 0.67 mM to 2.66 mM of As(III). (C) Results from planktonic cultures of CB1, CB2, CB3, CB6 and 3As grown with 1.33 mM of As(III). Two AioA are expressed for CB2 and CB3 and only one of similar weight for CB1, CB6, ACO3 and ACO7. (TIF) [file pone.0139011.s003.tif]

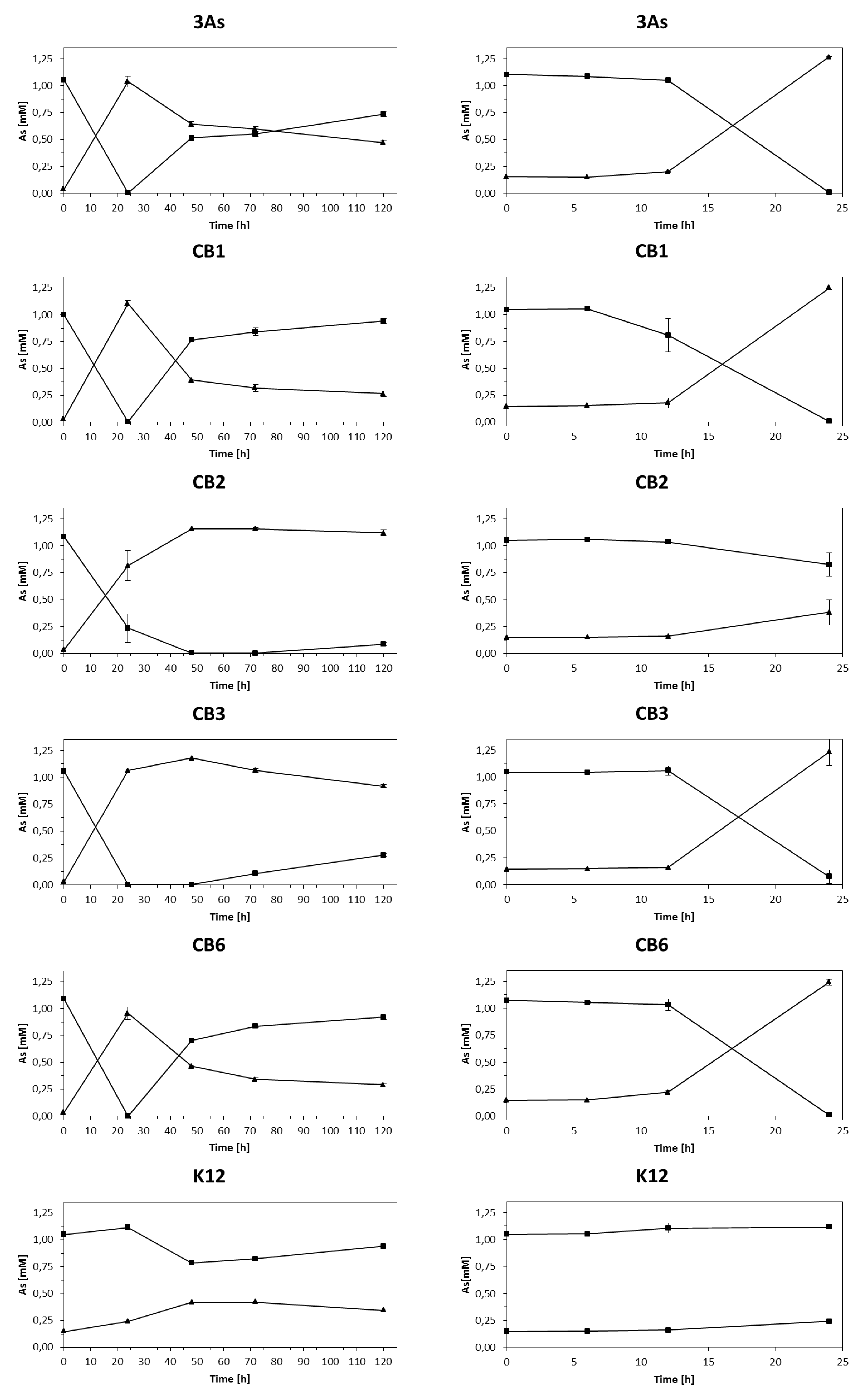

Supplement: S4 Fig — Concentrations of As(III) (squares) and As(V) (triangles) are shown for cells grown in m126 with an initial concentration of 1.33 mM As(III). On the left of the figure the concentrations measured at t0h, t+24h, t+48h, t+72h and t+120h are represented, while on the right the concentrations measured at t0h, t+6h, t+12h and t+24h are shown. Error bars indicate standard deviation of data obtained from triplicate cultures. The As(III) oxidation activity of K12 was lower than the strains from the AMD. After 24 h, and once all As(III) was oxidized, 3As, CB1, and CB6 reduced the As(V), which was not observed for CB2 or CB3. (TIF) [file pone.0139011.s004.tif]
