# Supplementary material for: Adaptation in Toxic Environments: Arsenic Genomic Islands in the Bacterial Genus Thiomonas
Source: PLoS One. 2015 Sep 30;10(9):e0139011. doi: 10.1371/journal.pone.0139011 (PMC4589449; doi:10.1371/journal.pone.0139011)
Supplement: S1 Table — Calculation of repetitive regions did not include undetermined (‘N’) bases. The genome size and gene content range from 3.4 Mb and 4, 367 CDS (K12) to 4.3 Mb and 4, 508 CDS (CB3). Thiomonas sp. CB3 also has a higher proportion of repetitive regions (19.21%). The others general features are similar between the strains. (DOCX) [file pone.0139011.s005.docx]

**S1 Table.** **General characteristics of the *Thiomonas* genomes.** Calculation of repetitive regions did not include undetermined (‘N’) bases. The genome size and gene content range from 3.4 Mb and 4, 367 CDS (K12) to 4.3 Mb and 4, 508 CDS (CB3). *Thiomonas* sp. CB3 also has a higher proportion of repetitive regions (19.21%). The others general features are similar between the strains.

| **Genome characteristics** | **CB2** | **3As** | **CB1** | **CB3** | **CB6** | **ACO3** | **ACO7** | **K12** |
| --- | --- | --- | --- | --- | --- | --- | --- | --- |
| Size (bp) | 3872510 | 3738778 | 3880244 | 4323494 | 3861602 | 3673425 | 3730618 | 3396378 |
| GC-content (%) | 63.67 | 63.8 | 63.8 | 63.93 | 63.83 | 63.8 | 63.78 | 63.88 |
| Predicted CDS | 3820 | 3631 | 3927 | 4508 | 3880 | 3628 | 3680 | 3467 |
| Genome coding (%) | 91.23 | 90.19 | 91.75 | 91.64 | 92.04 | 92.95 | 9181 | 90.11 |
| Average CDS size (bp) | 917.51 | 956.15 | 918.52 | 887.02 | 920.4 | 929.49 | 929.18 | 927.59 |
| Number of tRNA | 45 | 43 | 44 | 43 | 44 | 42 | 42 | 45 |
| ORF of unknown function  (%) | 1192  (31.20) | 939  (25.86) | 1238 (31,53) | 1514 (33.58) | 1212 (31.24) | 1097 (30.24) | 1130 (3071) | 218  (6.29%) |
| Repetitive regions (%) | 6.31 | 7.26 | 7.3 | 19.21 | 7.52 | 3.11 | 5.04 | 4.86 |
