# Supplementary material for: Adaptation in Toxic Environments: Arsenic Genomic Islands in the Bacterial Genus Thiomonas
Source: PLoS One. 2015 Sep 30;10(9):e0139011. doi: 10.1371/journal.pone.0139011 (PMC4589449; doi:10.1371/journal.pone.0139011)
Supplement: S2 Table — The RGP were identified with the RGP finder module in MaGe, which identify synteny breaks and search for HGT features (tRNA hotspots, genes involed in mobility), and for compositional bias (AlienHunter (Vernikos and Parkhill, 2006), SIGI-HMM (Waack et al., 2006), and GC deviation computation). (DOCX) [file pone.0139011.s006.docx]

**S2 Table. Regions of genomic plasticity (RGP) identified after comparison of CB2 with 3As and K12.** The RGP were identified with the *RGP finder* module in MaGe, which identify synteny breaks and search for HGT features (tRNA hotspots, genes involed in mobility), and for compositional bias (AlienHunter (Vernikos and Parkhill, 2006), SIGI-HMM (Waack *et al.*, 2006), and GC deviation computation).

| **RGP** | **Begin** | **End** | **Length** | **Feature Score** | **Feature** | **THIOM_3As** | **THIIK_K12** | **Specificity Score** | |
| --- | --- | --- | --- | --- | --- | --- | --- | --- | --- |
| RGP1 | 409 | 12446 | 12038 | 4 | tRNA-int-IVOM-Specific_Region | 71 | 71 | 142 | |
| RGP2 | 607500 | 625666 | 18167 | 1 | Specific_Region | 83 | 96 | 179 | |
| RGP3 | 750094 | 824454 | 74361 | 4 | int-SIGI-IVOM-Specific_Region | 88 | 70 | 158 | |
| RGP4 | 837543 | 916150 | 78608 | 3 | tRNA-IVOM-Specific_Region | 73 | 29 | 102 | |
| RGP5 | 1014685 | 1044464 | 29780 | 3 | SIGI-IVOM-Specific_Region | 93 | 93 | 186 | |
| RGP6 | 1111788 | 1130387 | 18600 | 2 | IVOM-Specific_Region | 82 | 71 | 153 | |
| RGP7 | 1188698 | 1206631 | 17934 | 0 | none | 13 | 100 | 113 | |
| RGP8 | 1393011 | 1428722 | 35712 | 4 | int-SIGI-IVOM-Specific_Region | 100 | 100 | 200 | |
| RGP9 | 1538398 | 1622571 | 84174 | 2 | misc_RNA-IVOM | 3 | 94 | 97 | |
| RGP10 | 1626740 | 1717910 | 91171 | 4 | int-SIGI-IVOM-Specific_Region | 72 | 27 | 99 | |
| RGP11 | 2196265 | 2202854 | 6590 | 1 | Specific_Region | 100 | 25 | 125 | |
| RGP12 | 2233184 | 2269464 | 36281 | 5 | tRNA-int-SIGI-IVOM-Specific_Region | 82 | 94 | 176 | |
| RGP13 | 2628929 | 2697519 | 68591 | 4 | tRNA-int-IVOM-Specific_Region | 52 | 77 | 129 | |
| RGP14 | 2794075 | 2816184 | 22110 | 3 | tRNA-IVOM-Specific_Region | 68 | 100 | 168 | |
| RGP15 | 2865731 | 2874024 | 8294 | 3 | tRNA-int-IVOM | 100 | 50 | 150 | |
| RGP16 | 2959834 | 2965781 | 5948 | 0 | none | 100 | 20 | 120 | |
| RGP17 | 3304256 | 3314146 | 9891 | 5 | tRNA-int-misc_RNA-IVOM-Specific_Region | 83 | 100 | 183 | |
| RGP18 | 3327601 | 3382919 | 55319 | 3 | int-IVOM-Specific_Region | 75 | 70 | 145 | |
| RGP19* | 3533461 | 3872209 | 338749 | 6 | tRNA-int-mob-SIGI-IVOM-Specific_Region | 50 | 69 | 119 | |
| * this island possibly corresponds to at least two distinct GEIs | | | | | | | | |  |
