# Supplementary material for: Adaptation in Toxic Environments: Arsenic Genomic Islands in the Bacterial Genus Thiomonas
Source: PLoS One. 2015 Sep 30;10(9):e0139011. doi: 10.1371/journal.pone.0139011 (PMC4589449; doi:10.1371/journal.pone.0139011)
Supplement: S5 Table — Thiomonas spp. CB2 and CB3 are slightly more resistant than the others strains. Thiomonas spp. 3As, CB1 and CB6 demonstrate lower levels of resistance to As(III). (DOCX) [file pone.0139011.s009.docx]

**S5 Table. Minimal inhibitory concentration (MIC) of As(III) for *Thiomonas* strains on solid m126.** *Thiomonas* spp. CB2 and CB3 are slightly more resistant than the others strains. *Thiomonas* spp. 3As, CB1 and CB6 demonstrate lower levels of resistance to As(III).

| ***Thiomonas* strains** | **K12** | **3As** | **CB1** | **CB2** | **CB3** | **CB6** | **ACO3** | **ACO7** |
| --- | --- | --- | --- | --- | --- | --- | --- | --- |
| **As(III) MIC (mM)** | 9.3 | 8 | 8 | 12 | 12 | 8 | 9.3 | 9.3 |
